# Impact of Cations and Framework on Trapdoor Behavior: A Study of Dynamic and In Situ Gas Analysis

**DOI:** 10.1021/acs.langmuir.4c00498

**Published:** 2024-06-04

**Authors:** Dankun Yang, Huan V. Doan, Una O’Hara, Daniel Reed, Julian Hungerford, Jean-Charles Eloi, Natalie E. Pridmore, Paul F. Henry, Sebastien Rochat, Mi Tian, Valeska P. Ting

**Affiliations:** † Department of Mechanical Engineering, University of Bristol, Bristol BS8 1TR, U.K.; ‡ Research School of Chemistry, Australian National University, Canberra 2601, Australia; § Department of Chemistry, University of Birmingham, Birmingham B15 2TT, U.K.; ∥ School of Metallurgy & Materials, University of Birmingham, Birmingham, B15 2TT, U.K.; ⊥ Micromeritics Instrument Corp., Norcross Georgia 30093, United States; # School of Chemistry, University of Bristol, Bristol BS8 1TS, U.K.; ∇ ISIS Pulsed Neutron & Muon Source, Rutherford Appleton Laboratory, Harwell Campus, Didcot, OX11 0QX, U.K.; ○ Department of Chemistry, Ångström Laboratory, Lägerhyddsvägen 1, Box 538, SE-751 21 Uppsala, Sweden; ◆ School of Engineering Mathematics and Technology, University of Bristol, Bristol BS8 1TS, U.K.; ¶ College of Engineering, Mathematics and Physical Sciences, University of Exeter, Exeter EX4 4QF, U.K.

## Abstract

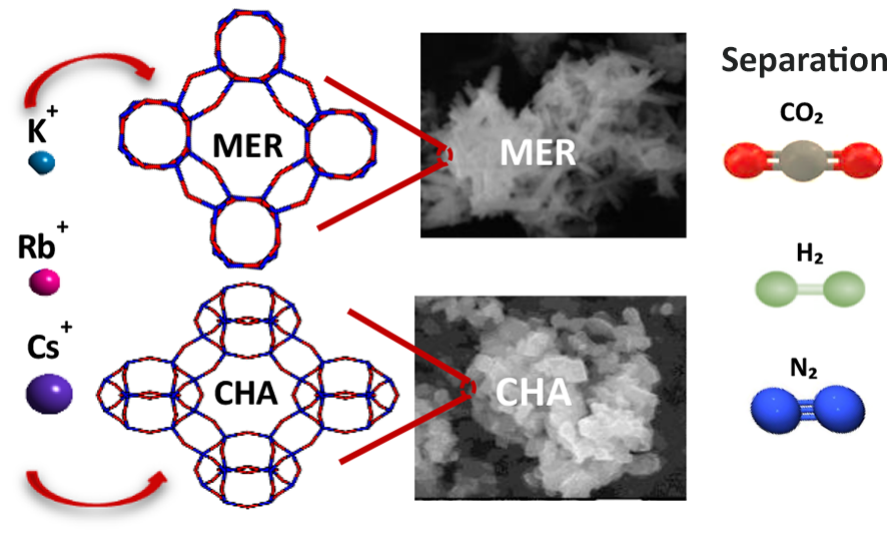

Due to their distinct and tailorable internal cavity structures, zeolites serve as promising materials for efficient and specific gas separations such as the separation of /CO_2_ from N_2_. A subset of zeolite materials exhibits trapdoor behavior which can be exploited for particularly challenging separations, such as the separation of hydrogen, deuterium, and tritium for the nuclear industry. This study systematically delves into the influence of the chabazite (CHA) and merlinoite (MER) zeolite frameworks combined with different door-keeping cations (K^**+**^, Rb^**+**^, and Cs^**+**^) on the trapdoor separation behavior under a variety of thermal and gas conditions. Both CHA and MER frameworks were synthesized from the same parent Y-zeolite and studied using in situ X-ray diffraction as a function of increasing temperatures under 1 bar H_**2**_ exposures. This resulted in distinct thermal responses, with merlinoite zeolites exhibiting expansion and chabazite zeolites showing contraction of the crystal structure. Simultaneous thermal analysis (STA) and gas sorption techniques further demonstrated how the size of trapdoor cations restricts access to the internal porosities of the zeolite frameworks. These findings highlight that both the zeolite frameworks and the associated trapdoor cations dictate the thermal response and gas sorption behavior. Frameworks determine the crystalline geometry, the maximum porosities, and displacement of the cation in gas sorption, while associated cations directly affect the blockage of the functional sites and the thermal behavior of the frameworks. This work contributes new insights into the efficient design of zeolites for gas separation applications and highlights the significant role of the trapdoor mechanism.

## Introduction

Zeolites, a class of porous materials with tunable pore sizes, are known for their ability to selectively separate gases based on molecular sizes.^1^ A subset of synthetic zeolites exhibits a unique characteristic whereby their pore openings can be thermally controlled, akin to a “trapdoor” mechanism. This feature has become a focus of interest in numerous studies on gas sorption and separation.^3^ The trapdoor behavior, significant for guest molecules of similar sizes, is particularly prevalent in functional pores bonded by Si, O, and Al (forming eight-membered rings, 8MR with size around 3.8 × 3.8 Å^2^ in chabazite framework structures).^2^ These functional pores are guarded by cations situated in the ring. This distinctive characteristic of trapdoor zeolites enables a novel form of molecular sieving. The process hinges on the ability of adsorbed guest molecules to cause a fully reversible deviation of the cation from the functional windows, thereby enabling selective entry of specific molecules.^3^ This unique thermal regulatory feature of trapdoor zeolites underlines their potential to provide sophisticated solutions for challenging gas separations.

Typically, gas molecules like H_2_ are restricted from entering the internal cavities of the zeolites unless sufficient thermal energy displaces the door-keeping cation.^4^ The required thermal energy is contingent on the framework types, cationic bonding content and the Si/Al ratio.^5^ In contrast, certain gas molecules, such as CO_2_,^6^ can bypass this requirement due to their large electronic quadrupole moment and high polarizability, allowing them to access internal pores without any increase in thermal energy.^7^

Chabazite frameworks (CHA, trigonal crystals with unit cell dimensions *a* = *b* = *c*)^8,9^ and merlinoite frameworks (MER, tetragonal crystals with unit diameter *a* = *b* > *c*)^10,11^ with trapdoor behavior are commonly studied for sustainable gas capture and separation due to their high microporosity and thermally controllable cavities with similar crystal sizes.^12^ The trapdoor mechanism in these zeolites has been explored for separating gases with close kinetic diameters, such as CO_2_, N_2_, and CH_4_,^13^ and even hydrogen isotopes such as H_2_ and D_2_.^14^ Such materials could find uses in future fusion energy applications. Zeolites with chabazite and merlinoite structures, both synthesized from the same parent zeolite under different alkaline levels^15^ or reaction periods,^16^ demonstrate high potential in gas separations but exhibit different thermal and gas response behaviors due to their distinct framework structures.

Apart from the framework, the trapdoor mechanism, which relies on the reversible cation displacement in the presence of various gases, necessitates the confinement of cations (such as K^+^ or other monovalent cations) within the windows, maintained by both electrostatic and van der Waals dispersion forces. This allows the frameworks to be simultaneously stable and tunable.^17^ Different cation sizes induce distinct behavior within the same frameworks. Webley et al.^3,18^ suggested that CHA frameworks with Rb^+^ and Cs^+^ exchanged cations showed better capacity in CO_2_/CH_4_ separation via the trapdoor mechanism compared to frameworks containing Li^+^ and Na^+^.^18^ Hong et al. also demonstrated that MER frameworks containing K^+^ and Na^+^ performed differently in CO_2_ sorption under the same conditions.^19^ Studies suggest that cations capable of fully blocking the 8MRs in the frameworks generate larger differences between open and closed trapdoor states,^8^ and the size and shape of the 8MRs can be tuned by creating frameworks with different diameters of the rings with different orientations. By controlling the position or direction of the functional window in which large cations can sit, different sorption behaviors can be obtained.

Therefore, the sorption properties of these zeolites are mainly affected by two factors: their framework structure and internal cations. The trapdoor framework structure dictates the crystal structure of the zeolite, thereby affecting the pore openings. The exchanged cations and their size,^20^ location,^21^ and valence^22^ further modulate porosity, sorption behaviors, and functionality of the trapdoor in zeolites.^23^ As a result, materials with distinct thermal behavior, hydrophilicity, and porosity can be obtained, resulting in finely tunable gas sorption behavior.^24^ Therefore, the highly adjustable structure and porosities of the trapdoor zeolites make them excellent candidates for various gas sorption applications.^25,26^

Merlinoite and chabazite are two types of zeolite frameworks with the potential of generating trapdoor behavior that can produced from the same parent zeolites with cation-accessible 8MRs.^27,28^ Herein, we focused on analyzing two factors in these two zeolites that can affect the behavior of the functional windows (8MRs) in gas sorption: the structure of the framework and different cations. The focus extends beyond analyzing the effect of different sizes of exchanged cations (K^+^ with a radius of 1.52 Å, Rb^+^ with a radius of 1.67 Å, and Cs^+^ with a radius of 1.81 Å)^29^ on CHA and MER frameworks, to include an examination of the impact of adsorbing different gases (H_2_, CO_2_, and N_2_) at various thermal conditions quantitively, dynamically and structurally. To further estimate the future application of the zeolites on gas separation or storage of different isotopes of hydrogen (some of which, like tritium, are radioactive) in the nuclear industry, radiation stability testing was also conducted to determine the robustness of the synthesized materials under exposure to radiation.

## Experimental Section

### Synthesis of Chabazite and Merlinoite

The synthesis of chabazite and merlinoite was performed following the method established by Kim et al.^30^ This process incorporated control of the dehydration process of the parent Y-zeolite (see the Supporting Information for details) followed by ion exchange.

### Ion Exchange

An amount of 1 g of parent CHA/MER was ion-exchanged with 1 M 40 mL of KCl (Merck Life Science, 98%), 1 M 40 mL of RbCl (Merck Life science, 98%), and 1 M 40 mL of CsCl (Merck Life science, 98%) at 70 °C, with stirring at 300 rpm for 24 h. The resulting product was then washed with 50 mL of deionized water using a centrifuge. This washing process was repeated at least three times, yielding KCHA, KMER, RbCHA, RbMER, CsCHA and CsMER. Successful ion exchange was confirmed by both PXRD and SEM.

### Characterization

#### Powder X-ray Diffraction (PXRD)

PXRD analysis was performed using a Bruker D8 Advance X-ray diffractometer in a flat plate geometry, employing a Cu Kα source, with wavelength λ = 1.5418 Å, spanning the range of 5°–60° 2θ, with a step size of 0.02 2θ at 293 K. In situ PXRD experiments were carried out on a D8 Advance X-ray diffractometer at the University of Birmingham. All measurements were accomplished on the samples synthesized at the same time and stored under the same atmosphere in a storage closet to maintain the same humidity. Prior to testing, samples were degassed in situ with a He flow at 473 K for 30 min and evacuated under vacuum conditions before changing the gaseous environment. Then the degassed samples were tested under 1 bar H_2_ from room temperature to 353 K (with increasing rate at 10 K min^–1^). Miller indices and unit cells from XRD results were analyzed using CrystalDiffract and CrystalMaker.

#### Scanning Electron Microscopy (SEM)

SEM images were captured at 15 kV and a working distance of 10 mm with an IT300 SEM instrument from JEOL, Japan. After mounting the samples onto a conductive carbon tape, they were coated with a thin layer of high-purity graphite (10–15 nm), using a Q150TES coater from Quorum Technologies Ltd., UK, to prevent electron charging and optimize characteristic X-ray collection. Energy-dispersive X-ray (EDX) data was collected alongside the SEM experiment using an X-Max 80 mm^2^ EDX detector and analyzed with AZtec software, both provided by Oxford Instruments, UK.

#### Simultaneous Thermal Analysis (STA)

STA was performed on around 15 mg of samples stored under the same lab environment (exposed to the same amount of humidity). Tests were performed under nitrogen gas flow (50 mL min^–1^), from room temperature (298 K) up to 773 K, with a heating rate of 10 K min^–1^ using a NETZSCH STA 449 F3 Jupiter instrument.

#### Inductively Coupled Plasma Optical Emission Spectrometry (ICP-OES)

Before analysis, a solid sample weighing 0.0195 g was dissolved in 7 mL of 2 M HCl at 40 °C, with stirring for 60 h. Following this, 0.1 mL of the resulting sample-acid solution was diluted with 9.9 mL of 1% nitric acid for comparison with the 10 ppm elemental standard. Subsequent measurements were conducted on an Agilent 710 simultaneous spectrometer using 10 mL of the diluted solutions and a blank containing 1% nitric acid. The system utilized Ar as the carrier gas, operating at a pressure of 5.5 bar with a purity of 99.998% (11 W, supplied by BOC), and a plasma gas flow of 1.5 L.

#### Gas Sorption and Breakthrough Experiments

Gas sorption was measured using a Micromeritics 3-Flex instrument from 0 to 1 bar at 77 K (controlled by liquid N_2_) and 273 K (ice bath) and 291 K (water bath), respectively. N_2_ (99.999%), CO_2_ (99.999%) and H_2_ (99.999%) used in the experiments were supplied by BOC. Before measurements, the samples were completely degassed under a high vacuum (10^–5^ mbar) at 473 K for more than 8 h to ensure comprehensive degassing without damaging the molecular structure, as confirmed by STA.

Pore size distribution was calculated based on the low-pressure gas sorption acquired on the 3-Flex instrument. This calculation involved fitting the adsorption branches of the isotherms to a slit-shaped pore model using the built-in program and models within the 3Flex MicroActive software. Specifically, density functional theory (DFT) was used for fitting the CO_2_ adsorption at 273 K, in the relative pressure p/p_o_ range from 10^–5^ to 0.03, to identify the nanopores below 10 Å with regularisation lower than 10^–4^. Nonlocal DFT (NLDFT) was used for the fitting of the N_2_ adsorption measured at 77 K, in the relative pressure p/p_o_ range from 0.05 to 0.9, to obtain the full range of the pores with regularisation lower than 10^–4^.

Breakthrough experiments with CO_2_ and H_2_ were performed on RbCHA (0.3 g) and RbMER (0.2 g) using a Micromeritics Breakthrough analyzer (BTA) at 298 and 373 K under 2 bar pressure. The sample powder was packed to a density of 1 g mL^–1^ in the middle of the cylindrical vertical breakthrough column (2.5 cm long and 1 cm^2^ in diameter) and fixed at either end using a 5A molecular sieve to ensure a consistent flow rate of the gas. A flow rate of 10 cm^3^ STP min^–1^ was set for the test gases, using He as the carrier gas at 25 cm^3^ STP min^–1^ and Ar as the signal gas at 5 cm^3^ STP min^–1^. A mass spectrometer measured the relative gas amounts in the column, detecting ion peaks at *m*/*z* = 40 for Ar, *m*/*z* = 44 for CO_2_, *m*/*z* = 4 for He and *m*/*z* = 2 for H_2_. Prior to the measurements, the samples were degassed at 473 K for 10 h under He flow to ensure complete dehydration.

#### Radiation Tests

Radiation stability testing was performed to estimate the stability of the zeolites under extreme conditions for the future gas separation or storage of hydrogen isotopes. The tests used a sealed, filtered Cs-137 source, generating 0.661 MeV γ-rays to replicate the type and flux of radiation that a sample might be exposed to when in an environment containing T_2_. Approximately 10 mg samples in open 5 mL glass vials were exposed to the γ-rays with a source-to-sample distance of 5 cm for over 190 h. Reference samples were stored under identical environmental conditions without exposure to a radiation source. Experiments were carried out in a controlled radiation environment at the ISIS Neutron and Muon Source.

## Results and Discussion

### Structure and Morphology

**Figure 1:**
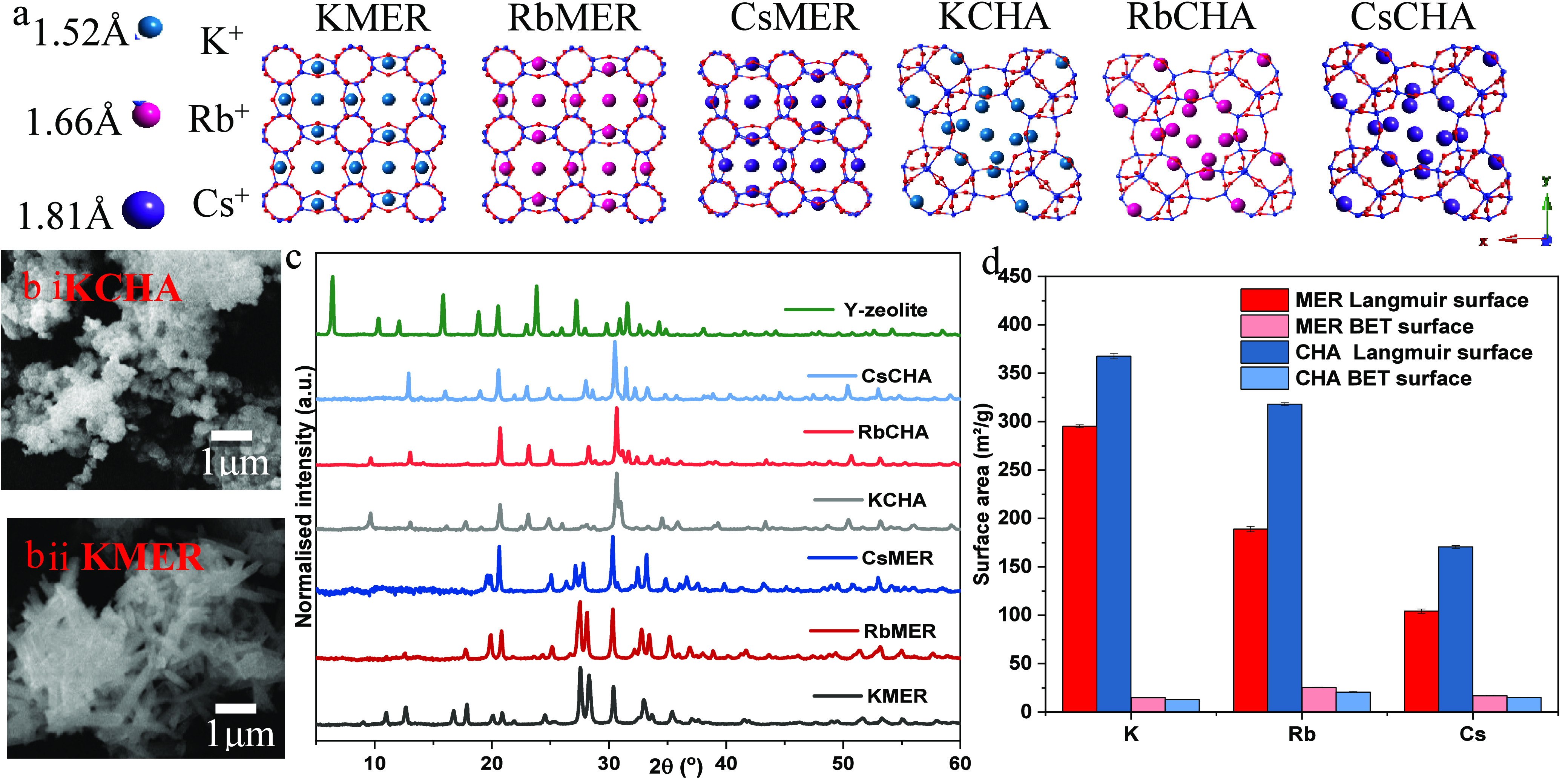
(a) Molecular structure of different MERs and CHAs (red and blue represent oxygen and silicon, respectively); (b) SEM pictures for KCHA (top) and KMER (bottom) as two representatives; (c) PXRD of parent Y-zeolite and synthesized chabazite and merlinoites; (d) Langmuir (red) and BET (light red) surface areas of merlinoites, and Langmuir (blue) and BET (light blue) surface areas of chabazite. Langmuir and BET surface areas were calculated from CO_2_ isotherms at 273 K and N_2_ isotherms at 77 K, respectively.

The synthesized samples were studied with SEM (detailed in Figure S1) and PXRD to characterize their morphology and crystalline properties (see Figure 1). In comparison to the trigonal crystals of chabazite (Figure 1b,i), the merlinoite crystals displayed preferential growth in a specific direction, leading to the formation of tetragonal structures (Figure 1b,ii). Characteristic peaks observed in the diffraction patterns indicated that both MER and CHA retained certain structural features from their parent Y-zeolites while simultaneously establishing unique unit cell sizes and crystal formations during the synthesis.

When heavier metal cations were introduced to the crystals, the patterns either shifted to higher 2θ (observed in the diffraction peaks of RbCHA and CsCHA in the angle range between 5 and 10° 2θ) or decreased in intensity, with some even disappearing completely (as seen in the diffraction peaks of RbMER and CsMER at 12° 2θ). The introduction of heavier cations appears to constrain the unit cell size, resulting in a tighter spacing between the crystal layers. This effect of cation size on unit cell size for both MER and CHA framework was previously demonstrated by Kong et al., specifically below 14° 2θ.^31^

Notably, the crystalline pattern shifts in the MER frameworks were mainly observed between 15 and 40° 2θ, whereas for CHA, the shift was concentrated in a narrower range from 25 to 35°. This implies that the crystalline structure of the MER frameworks is more sensitive to the size of the confined cations, which, in turn, has a direct impact on their porosity. Adsorption experiments using different gases can reveal variations in porosity due to the frameworks’ potential trapdoor behavior. Herein, both CO_2_ sorption at 273 K (Langmuir surface) and N_2_ sorption at 77 K (BET surface) were selected for surface area measurements. When assessing CO_2_ sorption at 273 K, chabazite displayed up to 55% greater Langmuir surface area than their MER counterparts, depending on the confined cation (Figure 1d). However, both crystal types had reduced surface areas with Cs^+^, the heaviest cation. Increasing the cation size consistently diminished the Langmuir surface area in both frameworks. Specifically, in merlinoites, the area decreased by 35.9% upon transitioning from K^+^ to Rb^+^ and dropped an additional 44.6% when switching Rb^+^ for Cs^+^. In contrast, chabazite exhibited a more linear decline at a gentler rate. To quantify, a 0.29 Å growth in cation diameter (*d*, *d*_K+_ = 3.04 Å, *d*_Rb+_ = 3.34 Å, and *d*_Cs+_ = 3.62 Å) resulted in approximately a 15% decrease in Langmuir surface area for chabazite. This is consistent with the observation that frameworks with larger cations exhibit tighter crystallographic spacing and reduced porosities, as indicated by the PXRD results.

**Figure 2:**
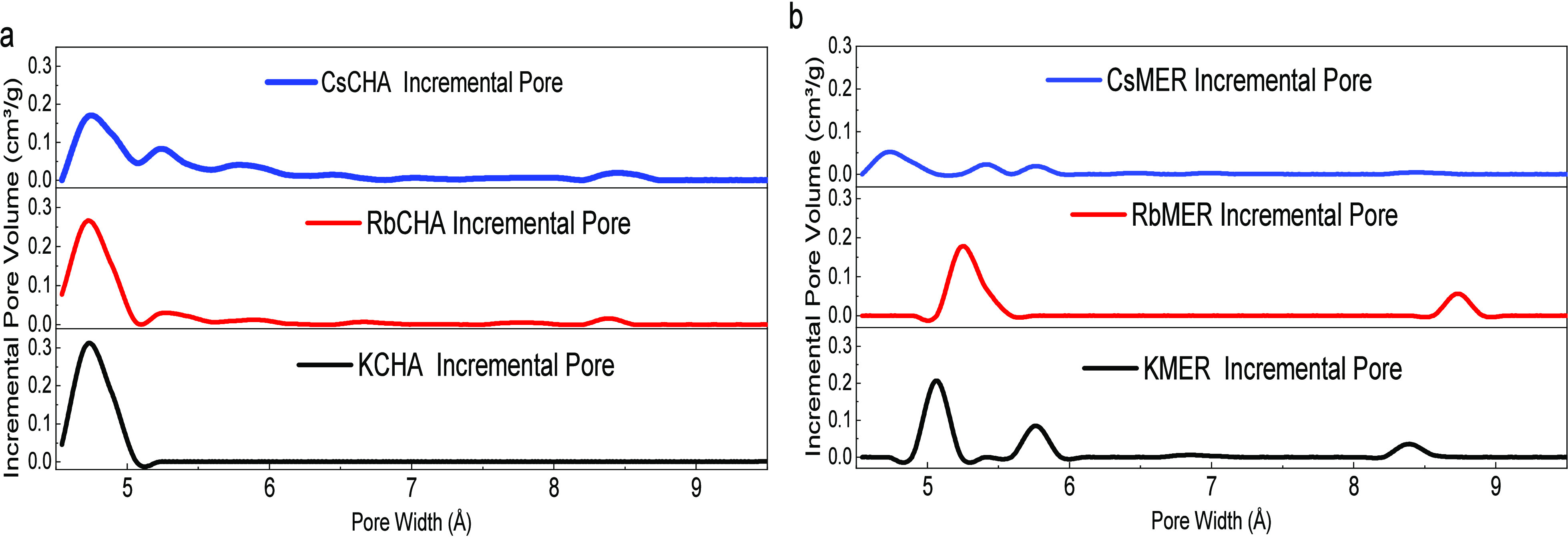
Pore size distribution calculated from DFT based on CO_2_ sorption at 273 K on (a) CHA frameworks with Cs^+^ (in blue), Rb^+^ (in red), and K^+^ (in black); (b) MER frameworks with Cs^+^ (in blue), Rb^+^ (in red) and K^+^ (in black).

All framework candidates showed comparable BET surface areas when measured with N_2_ at 77 K, but these values were significantly lower than those from CO_2_ isotherms (around 20 ± 5 m^2^g^–1^, in line with previous research by Doan et al.;^32^ further details can be found in Figure S2). The highest surface area was achieved by candidates with Rb^+^ (Figure 1d). Influenced by the size of the confined cations, the total pore volume for both frameworks decreased as the frameworks incorporated heavier cations (Figure 2). For CHA frameworks, the micropores primarily ranged between 4.5 and 5.0 Å, with the majority of pores centered at 4.7 Å.^31^ As the size of the confined cation increased, pores at 5.2 Å began to form on the CHA framework, likely due to the cation-induced expansion of the crystalline structure.^1^

Unlike CHAs, the impact of confined cations on MERs was distinct, stemming from their differing crystalline structures: the sizes of the primary micropores increased from 5.1 to 5.3 Å when the cation switched from K^+^ to Rb^+^, with similar behavior observed in pores at 8.3 Å (which increased to 8.6 Å). Some pores (around 5.8 Å) initially generated in KMER underwent modification by the larger cation (Rb^+^) and resulted in a single peak distribution from 5.1 to 5.6 Å.^33^ When the size of the confined cation further increased, the pore volume of merlinoites declined significantly, with a smaller pore size concentrated at 4.7 Å. Compared to MER frameworks, the pore size distribution of the CHA frameworks was more independent of the confined cations, likely due to the highly symmetrical crystalline geometry, consistent with the PXRD results. Together with the existing evidence of generated trapdoor behavior on Cs^+^ and Rb^+^ exchanged CHA and MER frameworks,^3,22,34^ the predilection for considerably higher measured CO_2_ surface areas hints at the existence of trapdoor behaviors for the synthesized candidates.

### Composition and Thermal Response

The elemental distribution obtained from EDX was presented and the ratio of different elements was calculated, as shown in Table 1. Despite originating from the same Y-zeolite, the Si/Al ratio for CHAs was approximately 2.3, while the Si/Al ratio for MERs was slightly lower when bound to the same cations. These suggest that MERs require a higher pH environment to attain the same Si/Al ratio as CHA frameworks.^28^ For both frameworks, the lowest Si/Al ratio was recorded for the Cs^+^ - containing structures. However, the Rb^+^ exchanged materials exhibited the highest Al (or Si) to cation ratio among the candidates (with more parent elements compared to the exchanged cation), indicating that under the same conditions, more Cs^+^ can be inserted within the zeolite frameworks compared to Rb^+^. All candidates displayed Al-to-cation ratios and Si/Al ratios within the range necessary for inducing trapdoor behavior.^18^

**Table 1:** Ratio of Framework Cation to the Exchanged Metal Cation for Chabazites and Merlinoites from EDX and Mass Loss of the Crystals during STA

	O atom %	Si atom %	Si/Al ratio	Si/exchanged ions	At% of exchanged cations	Mass loss % at 373 K	Mass loss % at 473 K	Mass loss % at 773 K
KMER	54%	24%	2.26 ± 0.06	2.38 ± 0.06	97%	1.8%	14.7%	19.6%
RbMER	60%	22%	2.13 ± 0.04	2.56 ± 0.04	94%	1.5%	9.7%	9.7%
CsMER	50%	22%	2.05 ± 0.04	2.18 ± 0.06	93%	1.4%	6.0%	6.7%
KCHA	55%	26%	2.29 ± 0.05	2.54 ± 0.02	94%	1.7%	12.5%	12.1%
RbCHA	58%	26%	2.35 ± 0.06	3.21 ± 0.15	84%	1.5%	11.1%	13.4%
CsCHA	54%	23%	2.28 ± 0.04	2.32 ± 0.24	86%	1.2%	9.3%	9.3%

Moreover, the atomic percentage of exchanged metal cations in the MER frameworks was consistently higher than in CHA frameworks (by 3% for K^+^-containing crystals, 10% for Rb^+^ -containing crystals, and 7% for Cs^+^ -containing crystals). This implies that the MER crystals were more receptive to cation exchange. The encapsulation of functional metal cations in both frameworks was slightly constrained by the purity of the starting salts and the efficacy of the exchange process but still formed high concentrations of the necessary functional cations. These results were backed by an ICP-OES analysis. Note that the ICP-OES results were limited by the weak emission energy detected from the Group I elements, with the signal from Cs^+^ being particularly weak and close to the wavelength of the Ar carrier gas.^35^ In addition, poor solubility of some zeolites in HCl can impede quantitative measurement.^36^ Nonetheless, ICP-OES showed clear enrichment of the cations in synthesized frameworks when comparing the starting materials (see Table S1).

**Figure 3:**
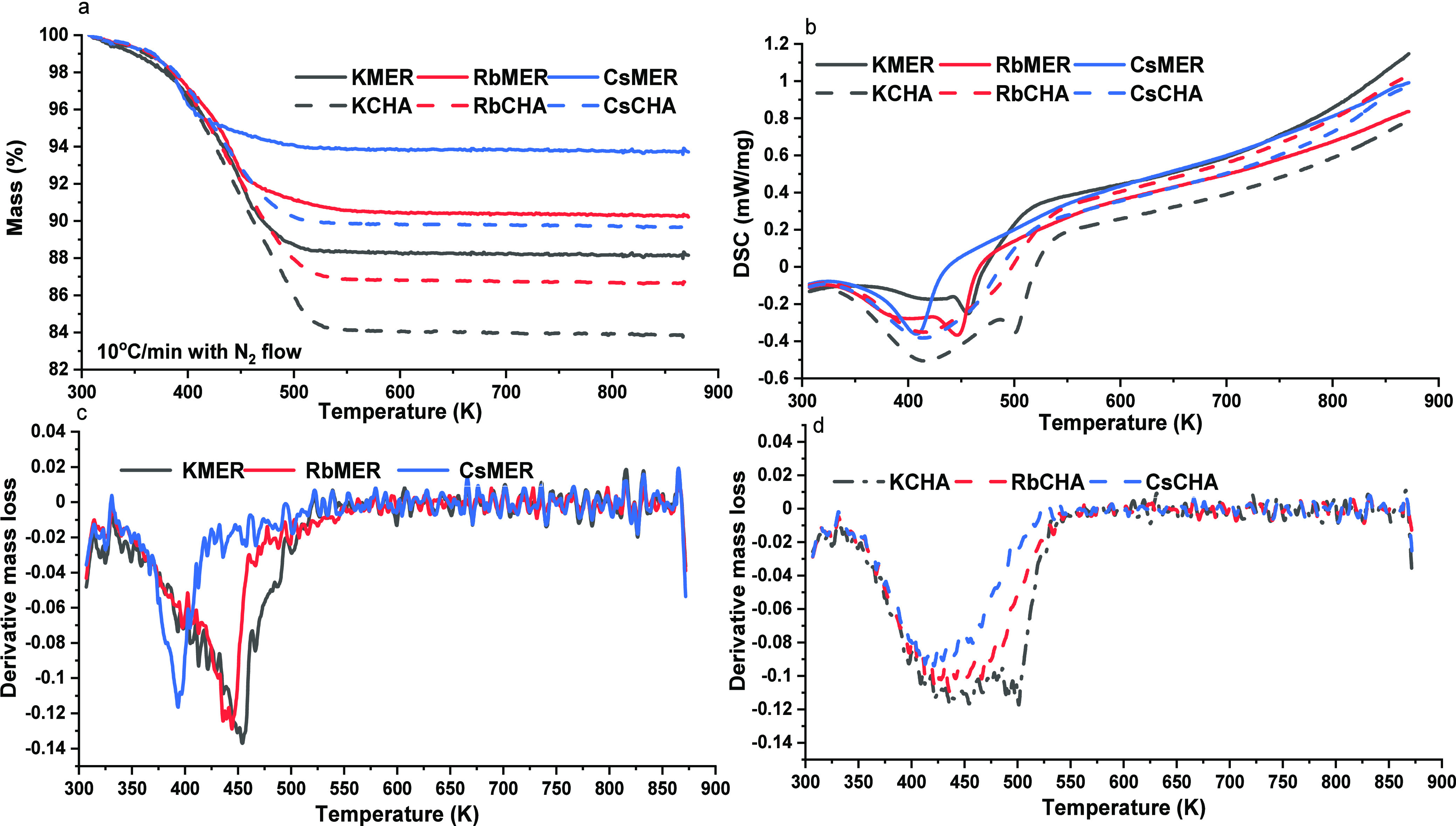
(a) TGA results for merlinoites and chabazites (black for K^+^, red for Rb ^+^, and blue for Cs^+^) up to 900 K under N_2_ flow; (b) DSC results for merlinoites and chabazites (black for K^+^, red for Rb ^+^, and blue for Cs^+^) up to 900 K under N_2_ flow; (c) mass loss derivative for MER frameworks (black for K^+^, red for Rb ^+^, and blue for Cs^+^); (d) mass loss derivative for CHA frameworks (black for K^+^, red for Rb ^+^, and blue for Cs^+^).

STA (up to 900 K, Figure 3) was performed on the candidates that were stored under the same conditions (exposed to the atmosphere) for over 8 weeks to generate an equal level of humidity. Results suggest that MER frameworks containing heavier (or larger) cations were less likely to hydrate, resulting in approximately 3% more residual mass (around 95% of the weight remaining for CsMER, 92% for RbMER, and 89% for KMER) after the experiment. A similar trend was observed in the CHA framework (around 84% remaining for KCHA, 87% for RbCHA, and 90% for CsCHA). In other words, the heavier cations block or occupy some of the pores, thereby limiting the surface area available for water molecules to occupy.

Another noteworthy aspect is that the mass of the candidates remained nearly constant at 373 K according to STA measurements, indicating that the water molecules are not freely mobile within the frameworks but are instead bonded to them and require a higher temperature for dehydration. When the temperature reached 473 K, the majority of the mass loss was achieved and further temperature increase (up to 900 K) did not result in noticeable changes. As confirmed by the PXRD results (Figure S3), the water molecules acted as crystal water (with 2θ angle mainly around 9°–13°and 27°–34°on CHA frameworks;^31^ 8°–13°and 31°–38° on MER frameworks^19^) and influenced the crystalline structure, implying that both zeolite crystals are sensitive to hydration, integrating H_2_O into their crystalline structure.^37,38^ Both DSC and mass derivative analyses indicate that the temperature at which maximum heat flow or mass loss occurs decreases with an increase in the cation size for both frameworks. Unlike chabazites, merlinoites display a narrower temperature range for mass loss with less heat transformation, suggesting a faster dehydration process relative to the more water-preferring chabazites.

Nonetheless, the temperature that triggers the maximum mass loss is consistent for the same cation-bonded CHA and MER frameworks, and the mass stabilizes above 473 K, which signifies the optimal temperature for removing crystal water in the frameworks. Furthermore, relative to CHA frameworks, MER synthesized over the same period exhibits a weight loss of approximately 5% higher and remains stable around 900 K with higher but stable heat flows during heating. In other words, MERs bonded with different cations experience lower mass loss at equivalent temperatures, denoting lower hydration levels compared to those of CHAs bonded with the same cations. This is corroborated by PXRD findings showing that the zeolites’ crystal structure remains intact after the STA process (Figure S4).

### Gas sorption Behavior and Breakthrough

**Figure 4:**
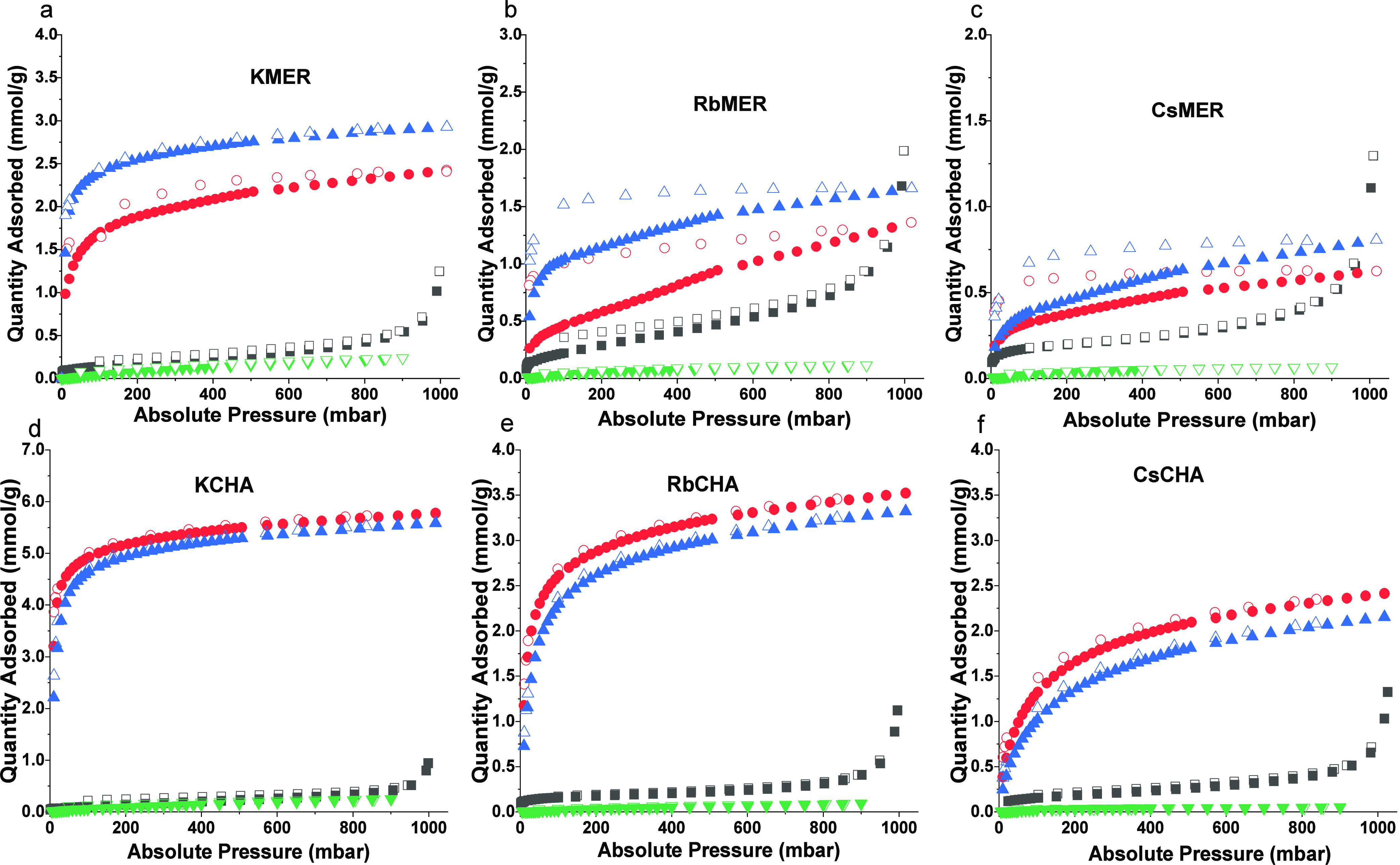
CO_2_ sorption at 273 K (red circles), CO_2_ sorption at 291 K (blue triangles), N_2_ sorption at 77 K (black squares), and H_2_ sorption at 77 K (green triangles) on (a) KMER, (b) RbMER, (c) CsMER, (d) KCHA, (e) RbCHA, and (f) CsCHA. Filled and empty symbols show adsorption and desorption branches, respectively.

The influence of the framework and cation on gas adsorption in the trapdoor system was evaluated by testing various gases with different molecular sizes and energy barriers^39^ (isotherms shown in Figure 4). H_2_ has the lowest uptake level at 77 K (highest value measured for KCHA at 0.25 mmol g^–1^, lowest value for CsCHA around 0.05 mmol g^–1^), particularly in comparison to CO_2_, which is favored due to its ability to penetrate the material’s interporosity by reducing the energy barrier through high adsorption enthalpy.

N_2_, a gas that behaves similarly to H_2_ but with higher uptake in the trapdoor system with Type II isotherm (nonporous or macroporous)^40^ indicating the gas molecules were prevented from entering the micropores, exhibits a relatively low sorption quantity (around 0.5 mmol g^–1^) at 77 K for all candidates. Different from other gases, the sorption of CO_2_ followed the typical Type I isotherm,^41^ suggesting CO_2_ is able to access micropores in the frameworks, proving the sorption pathways of CO_2_ differ from those of N_2_ and H_2_. Moreover, the level of CO_2_ uptake is significantly determined by the size of the bonded cations, as uptake is inversely proportional to the framework’s pore size. Specifically, uptake is nearly halved when the diameter of the cations increases by roughly 0.3 Å, with the order being K^+^ > Rb^+^ > Cs^+^.

Within the MER framework, elevating the adsorption temperature from 273 to 291 K leads to an increase in CO_2_ uptake, though the adsorbed amount decreases with the growth of contained cation size. Conversely, the quantity of CO_2_ adsorption in the CHA framework decreases with rising temperature. Simultaneously, CO_2_ uptakes decrease with the increasing size of the integrated cations. This indicates that adsorption onto the intermicropores of the MER frameworks with different functional window structures requires higher thermal energy to propagate, while the micropores in the CHA frameworks can be more easily filled at lower temperatures showing high potential in applying for CO_2_ storage.

In contrast to CHA frameworks, which can smoothly desorb the CO_2_ gas molecules, the adsorbed gas molecules in MER frameworks are more likely to be trapped within the doors and become harder to desorb due to the perpendicularly oriented functional rings. The observation of hysteresis on the CO_2_ sorption isotherms of merlinoite confirms that the molecules can pass through the functional windows (8MR sites). The desorption of CO_2_ required much lower pressure to pump out the molecules, potentially indicating the adsorbed CO_2_ molecules propagated into merlinoite cells among the longest α direction (around 13.5 Å),^19^ where most 8MR sites are located. This desorption behavior is specific to Rb^+^ and Cs^+^ containing merlinoites; K^+^ ions may be too small to fully block the “trapdoor” sites in MER frameworks, allowing adsorbed gas to be removed more freely. The size of the cation directly influences the blockage of the functional windows, impacting trapdoor performance. When the trapdoor is unobstructed, MER frameworks exhibit a benefit in CO_2_ capture. Therefore, to ensure optimal trapdoor functionality, the chosen cation should have a size comparable to the 8MR windows in the framework.

The differing adsorption processes of gases within the trapdoor system can be more directly understood through breakthrough experiments with gas access to different pathways in the zeolite system such as CO_2_ and H_2_. Gases like CO_2_ can diffuse through the trapdoor at all temperatures by reducing the potential well of door-keeping cations in the gas molecule side of the functional doors.^42−44^ Conversely, gases like H_2_ are unable to pass through the trapdoor window under a certain threshold temperature, thus creating highly suitable conditions for observing different dynamic behaviors of gas molecules in breakthrough experiments. Rb^+^ exchanged frameworks (RbCHA and RbMER^10,45^) were chosen as they displayed relatively high uptakes of CO_2_ compared to Cs^+^ exchanged frameworks. Prior literature reports also indicate that gas uptake at higher temperatures (348 K) is more likely to be observed in Rb^+^ exchanged frameworks than in frameworks containing K^+^ which can block the functional windows.^19,46,47^

**Figure 5:**
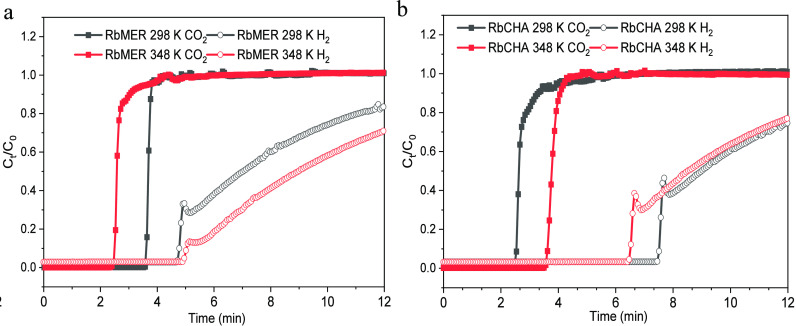
Breakthrough curves of (a) CO_2_ (black squares), H_2_ (black hollow circles) at 298 K and CO_2_ (red squares), H_2_ (red hollow circles) at 348 K on RbMER; (b) CO_2_ (black squares), H_2_ (black hollow circles) at 298 K and CO_2_ (red squares), H_2_ (red hollow circles) at 348 K on RbCHA.

To observe the effects of the threshold temperature, frameworks exchanged with the same cations were tested at 298 K (room temperature) and 348 K (the highest recorded threshold temperature for the typical trapdoor materials on N_2_).^48^ The breakthrough curves of CO_2_ and H_2_ are shown in Figure 5, with the full experimental process can be found in Figure S5. For the Rb^+^ bonded MER framework, the breakthrough time of CO_2_ (the time from breakthrough start to fully stabilized is approximately 3.7 min) remains consistent at both 298 and 348 K. A similar time frame is observed in the same cation-bonded CHA framework at 298 K. This suggests that raising the temperature does not result in kinetic differences in the MER system. However, the propagation time of CO_2_ through the CHA sample slightly increased from 4.2 to 4.6 min when the temperature was raised to 348 K. This difference is likely attributable to variations in the crystalline structure of the framework. Compared to the tetrahedral crystalline structure of the merlinoite, the trigonal crystalline structure of the chabazite provides more entry points for CO_2_ to explore once the door-keeping cation has been displaced due to a high thermal energy supply, thereby resulting in a longer breakthrough time.

Similar to the behavior observed on CO_2_ in the RbMER system, the pass-through time of H_2_ in both the RbMER and RbCHA systems appears to be unaffected by the increase in temperature. This is likely because the uptake amount of H_2_ is quite limited, as demonstrated in Figure 5, and, hence, fails to create a noticeable dynamic difference. When compared to the MER framework, the intensity detected by the CHA framework is higher at the outset, indicating that less H_2_ remains within the RbCHA system in comparison to the RbMER system (additional details, along with the derivative of weight change, are illustrated in Figure S6).

Although CO_2_ takes a longer time to traverse RbCHA with an increase in temperature, the calculated adsorbed amount of CO_2_ decreases from 20.1 cm^3^ g^–1^ STP (298 K) to 17.9 cm^3^ g^–1^ STP (348 K). Constrained by porosity and surface area, the uptake of CO_2_ in the RbMER system is 40% (8.0 cm^3^ g^–1^ STP at 298 K) of that of RbCHA. However, the final uptake slightly increases to 10.1 cm^3^ g^–1^ STP when the temperature is raised to 348 K. This demonstrates that an increased external thermal energy supply can aid the MER frameworks in adsorbing CO_2_, which aligns with results derived from the low-pressure sorption.

Contrary to CO_2_, which displayed a distinct breakthrough curve, the results for H_2_ (details depicted in Figure S6) suggest that it tends to pass through the sample without producing discernible uptakes (<3 cm^3^ g^–1^ STP). For both MER and CHA frameworks, the gas intensity of H_2_ diminishes by around 0.3 cm^3^ g^–1^ STP when the temperature increases from 298 to 348 K, implying a marginal increase in H_2_ uptake at 348 K. In essence, the measurement temperature exceeds the threshold temperatures of H_2_ in both systems, which results in only a slight uptick in H_2_ uptake at 298 K. To confirm the uptick of the H_2_ was caused by the opening of the trapdoor, in situ PXRD under a H_2_ environment was measured.

### In Situ Analysis of Crystalline Behavior Induced by Gas

**Figure 6:**
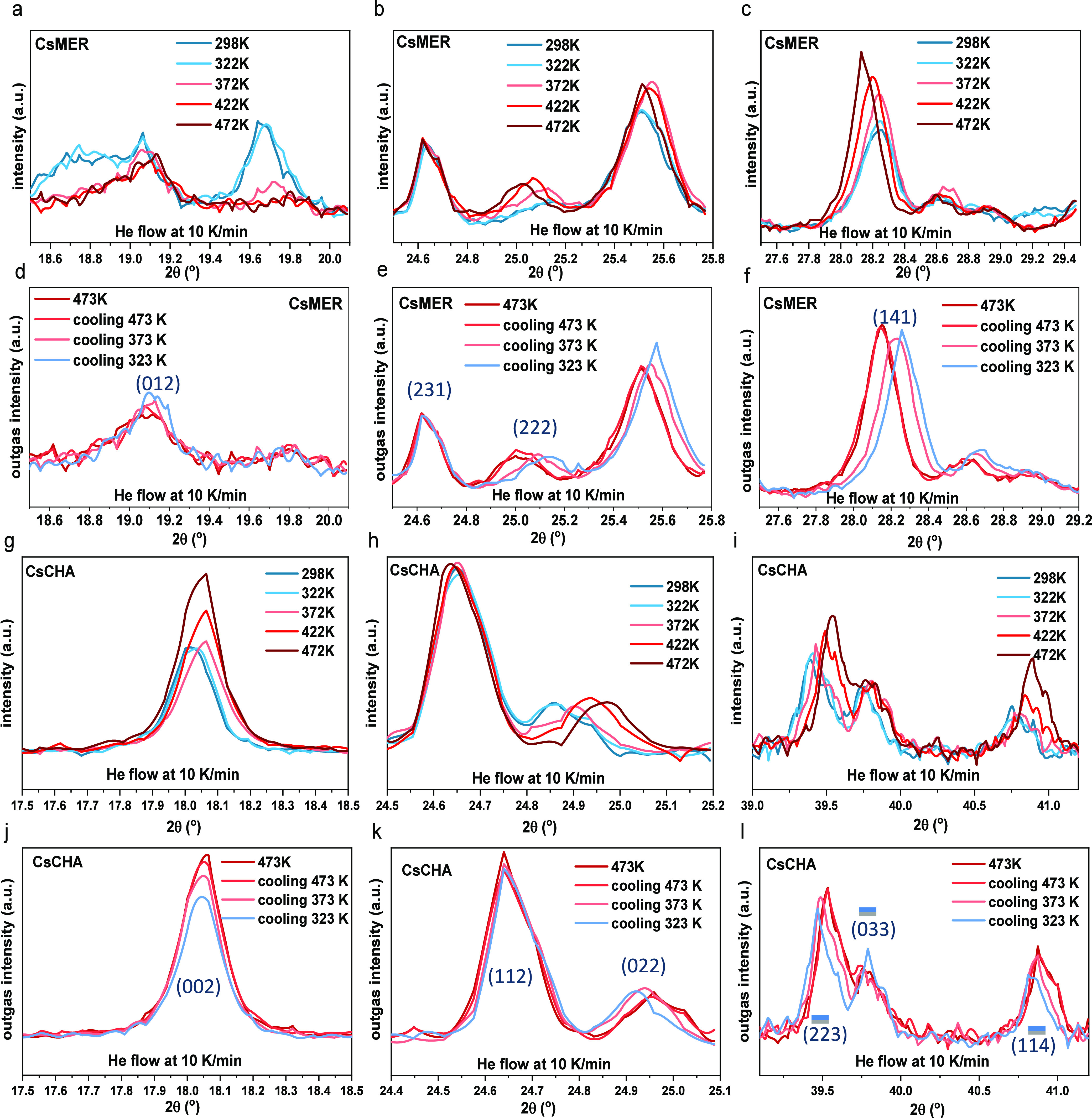
In situ degassing process on CsMER at 2θ angle (a) 17.5°–20.2°; (b) 24.3°–26.0°; (c) 27.5°–30.0° (298 K in deep blue, 322 K in light blue, 373 K in pink, 422 K in red, and 472 K in dark red); in situ cooling down process with He flow of CsCHA at 2θ (d) 17.5°–20.2°; (e) 24.3°–26.0°; (f) 27.5°–30.0° (473 K in deep red, cooling down 473 K in red, 373 K in pink, and 323 K in sky blue); in situ degas process on CsCHA at 2θ angle (g) 17.5°–18.5°; (h) 24.3°–25.5°; (i) 38.7°–41.5° (298 K in deep blue, 322 K in light blue, 373 K in pink, 422 K in red, and 472 K in dark red); in situ cooling down process with He flow of CsCHA at 2θ angle (j) 17.5°–18.5°; (k) 24.3°–25.5°; (l) 38.7°–41.5° (heating up 473 K in deep red, cooling down 473 K in red, 373 K in pink, and 323 K in sky blue).

In situ PXRD can also offer a view of the interaction between the adsorbed gas^49^ (H_2_, as shown in Figure 6) and the frameworks. The results were recorded every 10 K during the temperature increase (298–472 K) and every 50 K when decreasing temperature (473–323 K) on Cs^+^ confined frameworks to estimate the reversibility of changes in the crystalline structure under certain thermal conditions. The Cs^+^ exchanged framework was chosen to explore the thermal impact on the gas sorption as former research confirmed all exchanged Cs^+^ was sitting on the most energetically favorable sites (SIII′, 8MR) for chabazite with Si/Al ratio around 2.5 at below 333 K with only 5% migration,^23,17^ which maximizes the occupation of the functional window and highly limits the thermal effect below the threshold temperature.

Therefore, these frameworks are more likely to undergo potential temperature-dependent effects caused by adsorbed H_2_. Figure 6 (with the full angular range in Figure S7) demonstrates that when Cs^+^ exchanged frameworks were heated to 473 K under He flow and cooled to 323 K, both reversible (thermal structural response)^50,51^ and irreversible (loss of crystalline hydration structure) transformations were observed. Aligning with Bragg’s law,^52^ elevated thermal energy caused a reversible expansion of the merlinoite crystal layer distance, indicating a larger grain size. Notably, certain low-angle peaks vanished in merlinoite (19.6°, (012), Figure 6a) due to the lack of crystal water in the *ste* building units.^19^ This occurred as the cation (Cs^+^), expelled from the window by the H_2_O in the hydrated frameworks, moved back to the window center after dehydration.^19^ Simultaneously, some other features from the *d8r* building unit, previously obscured by moisture, became visible postdehydration (25°, (222), Figure 6b) or exhibited increased intensity (25° to 29°, (040), Figure 6c), emphasizing the influence of moisture on crystalline behavior.

Upon complete dehydration, the unit cells contracted during the cooling process, with the characteristic peaks shifting to the higher 2θ values (as shown in Figure 6e and f with the peak at around 25.5 and 28.5°) due to the shrinkage of the frameworks. However, the increased intensity due to the enlarged grain size during the dehydration process remained unaffected (Figure 6c and f). In contrast, the elevated thermal energy contracted the distance between chabazite layers (022), causing the diffraction peaks to shift toward larger diffraction angles during the dehydration process (24.8°–25.3°). Similar to merlinoite, the increased intensities and larger grain size, attributable to gained thermal energy, reversed back with decreasing temperatures (18°, (002), Figure 6 and j), demonstrating the high thermal tolerance of the crystal structure. The reversible expansion or contraction of the atomic layers resulted in a reversible change in crystal grain size, indicating that the candidates are suitable for multiple gas sorption cycles at temperatures up to 472 K without irreversible effects on their crystalline structures.

The fully dehydrated frameworks were exposed to 1 bar of H_2_ at the measured temperatures (303–362 K, the full range shown in Figure S8). Predictably, the *d*-spacing between the porous crystalline layers, as shown in the diffraction pattern of the frameworks, contracted due to the physical sorption process incited by increased thermal energy.^53,54^

**Figure 7:**
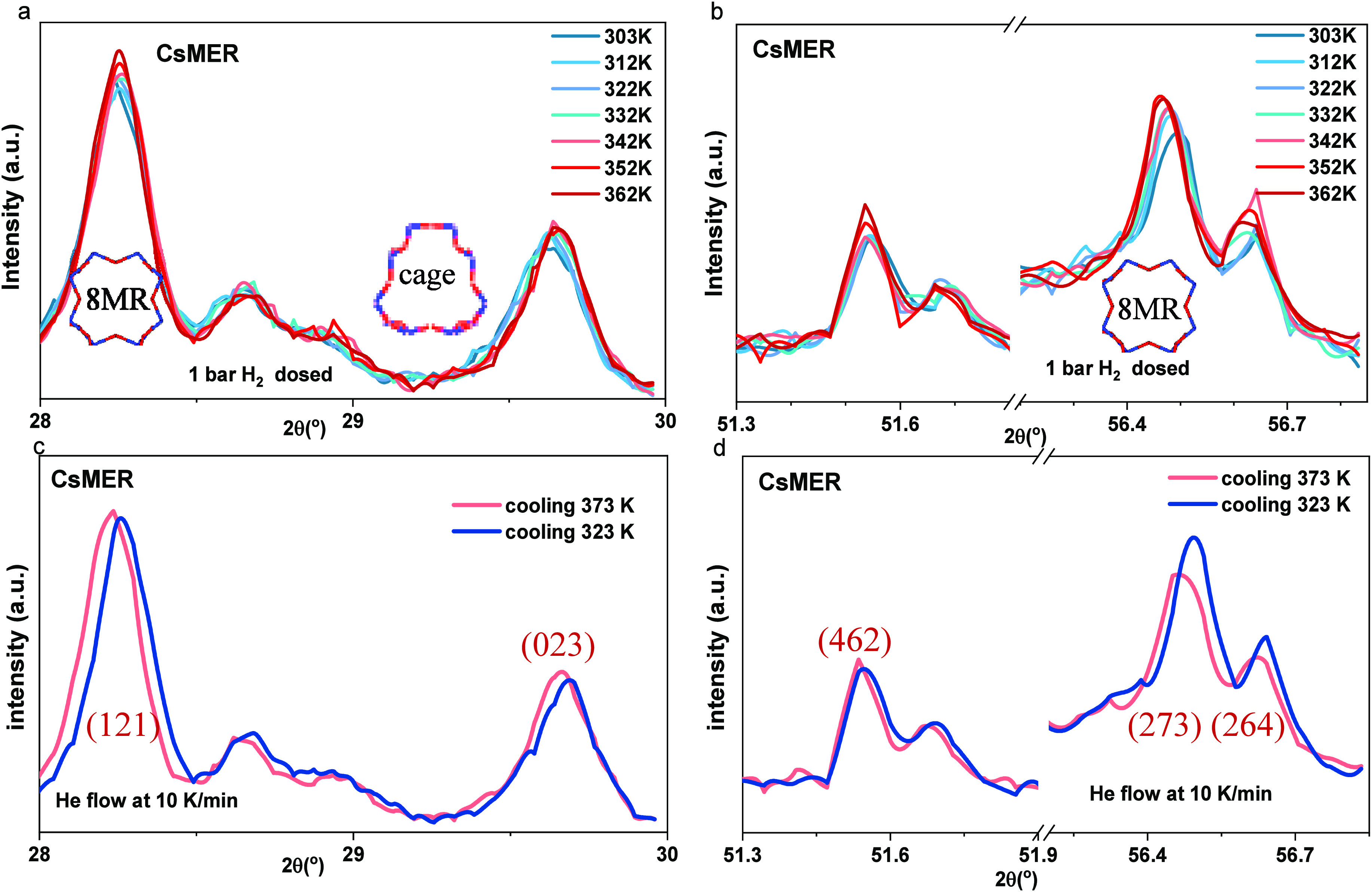
In situ PXRD with temperature increased from 303 to 372 K with 10 K increase per measurement for CsMER dosed with 1 bar H_2_, showing 2θ from (a) 28° to 30°; (b) 51.3° to 56.8°. Thermal effect on CsMER from 373 to 323 K through the outgas process, showing 2θ from (c) 28°–30°; (d) 51.3°–56.8°.

By comparing the thermal effects observed on the dehydrated frameworks during the cooling process under He flow with the frameworks exposed to 1 bar of H_2_, the influence of the H_2_ can be determined. The H_2_-dosed CsMER shows an increase in intensity with a lower half-width without peak shifting (Figure 7a, 28.3°, (121)) or even shifting to the higher range (Figure 7a, 30°, (023)) suggesting the adsorbed gas molecules extend the grain size of the functional sites by displacing the center cation toward the edge of windows (Figure 7a, 28.3°) and squeezed the spacing between the cage (6MR) sites (Figure 7a, 30°). Similar behavior can be observed at a specific diffraction angle of CsMER (Figure 7b, 51.6° (462) and 56.4° (273)).

However, without H_2_ dosing, the diffraction pattern shifts to the lower diffraction angle on the cage sites (expansion of the *d*-spacing) with the thermal effect (Figure 7c, 28°–30°). The thermal effect from temperature increase (Figure 7d) suggested a clear intensity decrease with a wider half-width level and a slight shifting to a higher 2θ due to shrinkage of the frameworks. In contrast, in the presence of H_2_, these peaks displayed an increased, broadened intensity. That indicates the adsorbed H_2_ had a much stronger effect on the framework when the temperature increased, leading to the expansion of the grain size as the gas molecules occupied the windows of the frameworks. This expansion increased with temperature, suggesting that higher thermal conditions permitted more H_2_ to be adsorbed in the functional sites with the displacing of the center cation (Cs^+^) and increasing grain size. At temperatures above 352 K, a pronounced increase in peak intensity with a lower half-width was observed in CsMER.

**Figure 8:**
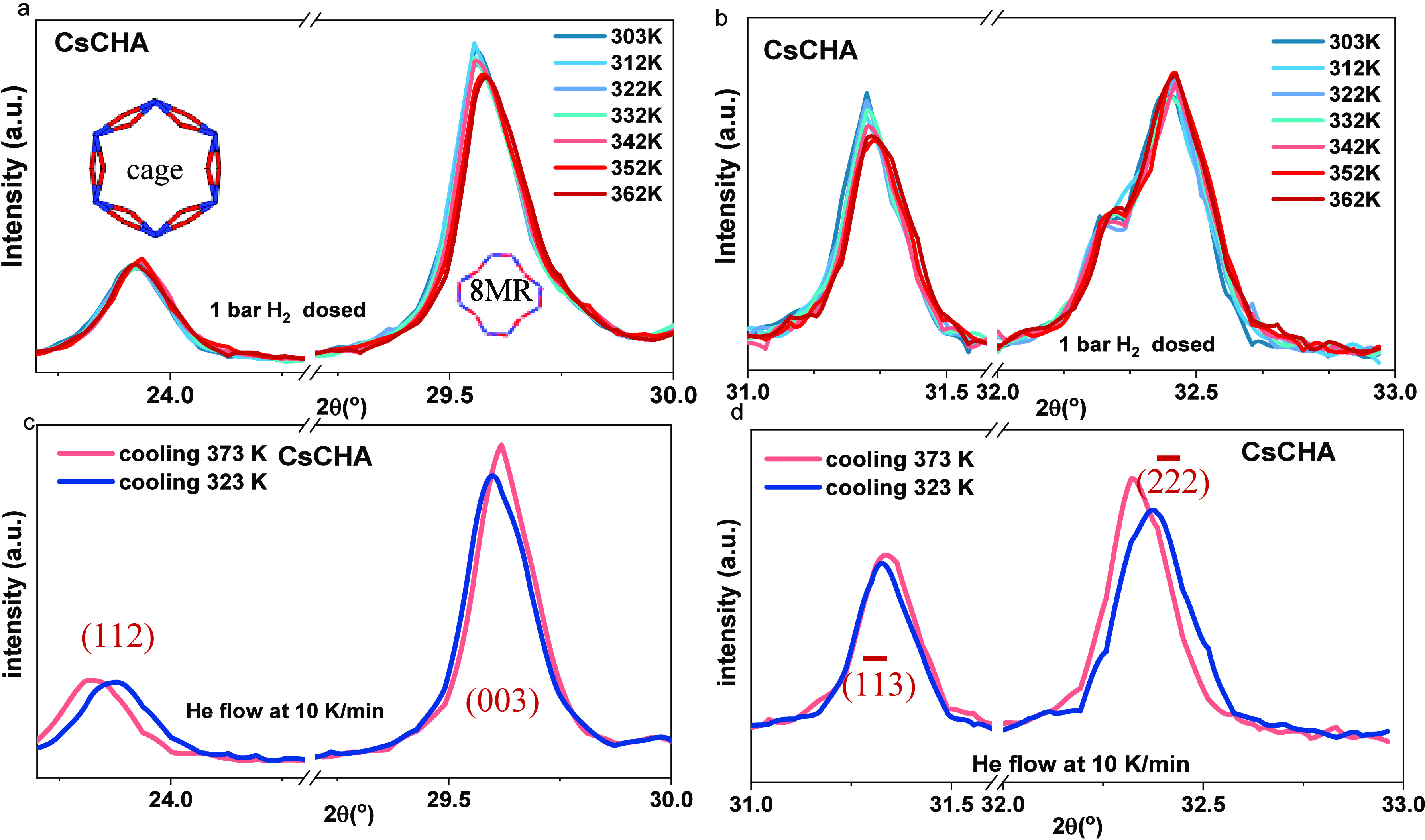
In situ 1 bar H_2_ dosed PXRD with temperature increased from 303 to 372 K with 10 K increase per measurement for CsCHA, showing 2θ from (a) 23.5° to 30°; b) 31° to 33°. Thermal effect on CsCHA with 2θ from 373 to 323 K through the outgas process at (c) 30°–31.5°; (d) 33.5°–37°.

The interaction between the framework and adsorbed H_2_ can also be observed on the crystal structure of chabazite, but in a different way. The adsorption impact on the cage sites and functional windows is distinguishable since the response of the functional windows in CHA is affected by the temperature increase (Figure 8a, 29.6° (112); Figure 8b, 31.3° (003)). When the temperature increased close to and gradually above the threshold temperature, more functional windows started adsorbing H_2_. Therefore, the peak intensity of the neighbor sites (6MR) of 8MRs decreased with the temperature increase, leading to a broad peak with a smaller grain size when the cation was pushing toward the neighbor sites, while the thermal response to temperature increase resulted in an intensity increase (Figure 8c, 29.6° (11̅3); Figure 8d, 31.3°).

For the cage sites inside CHA in which sorption capacity is not dependent on temperature, gas sorption at different temperatures did not result in different peaks (Figure 8a, 23.9°; Figure 8b 32.5°) but restricted the *d*-spacing between the crystalline layers shown as preventing the peak shifting to the smaller diffraction angle due to thermal effect. A peak broadening due to gas adsorption in the cage sites can be observed at 32.3° with a slight intensity increase, and shifting to a higher diffraction angle can be noticed on the neighbor peak (32.5° Figure 8b (22̅2)), showing the neighbor sites of the functional window decreased in *d*-spacing due to the extrusion of the gas-absorbed functional sites. The slight shift and increased intensity observed upon H_2_ adsorption can be attributed to the gas sorption behavior.

A significant reduction in peak intensity is noted at temperatures exceeding 352 K, suggesting that the critical temperature range for H_2_ trapdoor opening in CsCHA lies between 342 and 352 K. Results for merlinoites and chabazites indicate that the trapdoor for H_2_ becomes accessible within this temperature range, underscoring the pivotal role of metal cations in regulating trapdoor thermal response. The influence of temperature on the framework structure was further influenced by H_2_ sorption, indicating that the presence of H_2_ induces additional changes beyond the response to temperature variations in the crystalline framework. The effects observed upon temperature increase can be attributed to guest molecule adsorption at specific sites, with dosed H_2_ primarily affecting the 8MR sites during the temperature increase.

### Radiation Stability

To explore another potential application of the zeolites with trapdoor behavior (separating gases of radioactive isotopes like T_2_ from H_2_), the medium-term stability of these trapdoor materials was investigated.

**Figure 9:**
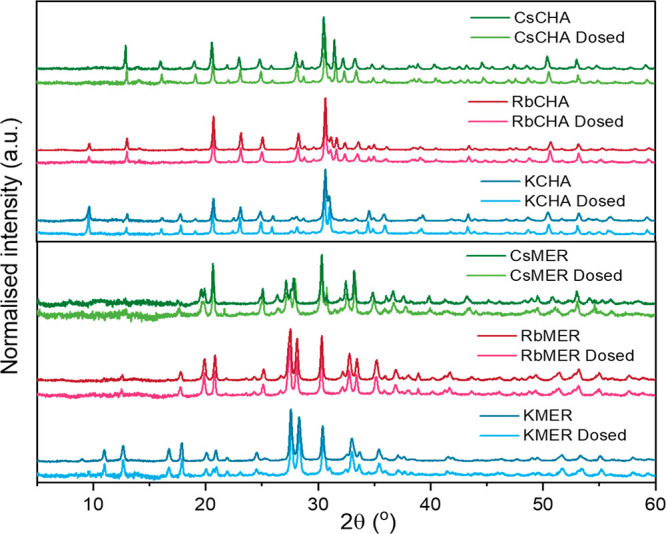
Powder X-ray diffraction results of the control zeolite samples and the Cs-137 (γ-ray) dosed samples.

As can be seen from the PXRD results in Figure 9, both merlinoites and chabazites showed solid stability under γ-ray exposure (2 Sv/h) for over 1 week with no substantial changes to their crystalline structures. In addition, SEM imaging showed no significant changes or crystalline deformation caused by radiation damage after dosing (see Figures S9, S10).

Compared to He or H_2_, T_2_ is more likely able to access the internal capacities of the functional windows (8MRs) by lowering the energy barrier of the door-keeping cation with higher dipole moments and polarization.^34^ Therefore, due to the high tolerance of these materials to radiation, the separation of T_2_ may be achieved by exploiting the difference in accessible capacities of these trapdoor zeolites without damaging the crystalline structure, even after long exposure.

## Conclusion

This study provides a comprehensive investigation of the influence of zeolite trapdoor frameworks (CHA and MER) and door-keeping cations (K^+^, Rb^+^, and Cs^+^) on gas adsorption and thermal behavior. The trapdoor framework structure dictates the size of the pore openings and the crystalline behavior. The exchanged cations are responsible for further restricting the porosity, sorption behaviors, and functionality of the trapdoor of the zeolites.

Both framework types exhibit excellent reversibility in heat treatment, but the loss of crystalline hydration and interaction with the adsorbed guest molecules can trigger different structural responses in merlinoite and chabazite. The framework structure of merlinoite is more flexible and shows an expansion in the pore size at high temperatures, while chabazite has a more rigid structure and shows a tightening of the pore size. Compared to the thermal effect, the crystalline structure will primarily respond to the adsorbed gas molecules, and the effect of threshold temperature can be generated on the functional sites, as discovered from in situ PXRD.

The gas adsorption behavior suggests that both the framework and bonded cation can influence the sorption amount and the crystalline response in gas sorption. Increasing temperature can increase the quantity of CO_2_ adsorbed by MER frameworks, but the opposite trend was observed for the CHA frameworks, showing the difference in the geometry of the functional windows can result in different requirements of thermal energy to open up the full microporosities. Nonetheless, to make sure the trapdoor sites can be fully functional, the chosen cation should have a comparable size to the 8MR windows in the framework (Rb^+^ for MER framework, Cs^+^ for CHA framework).

Compared to chabazites, merlinoites exhibit a greater CO_2_ capture capacity near room temperature and an incomplete desorption. Increased thermal energy prolongs the CO_2_ breakthrough time in the CHA framework and seems to boost uptake in MER frameworks. H_2_ shows minimal dynamic adsorption, indicating direct passing of gas through the CHA framework. Thus, owing to their higher surface areas, chabazites would be more applicable for static gas storage or separation, whereas merlinoites, which display strong temperature dependence of their gas uptake, are promising for gas capture. Moreover, the outstanding radiation stability of both types of trapdoor zeolite further enriches the application of trapdoor mechanisms in gas separation.

## Supporting Information Available

Synthesis of chabazites and merlinoites; SEM images of all zeolites; elemental concentration from ICP-OES; pore size distribution of the materials; unit cell volume of materials; PXRD of samples before and after degassing and STA; detailed breakthrough results on zeolites; full range in situ PXRD for candidates during degassing and H_2_ sorption; radiation stability of the zeolites (PDF)

## Abbreviations

CHA: chabazite
MER: merlinoite
STA: simultaneous thermal analysis
PXRD: powder X-ray diffraction
SEM: scanning electron microscopy
DSC: differential scanning calorimetry
8MR: eight-membered rings
6MR: six-membered rings
ICP-OES: inductively coupled plasma optical emission spectrometry
