# Supplementary material for: Impact of Cations and Framework on Trapdoor Behavior: A Study of Dynamic and In Situ Gas Analysis
Source: Langmuir. 2024 Jun 4;40(24):12394–406. doi: 10.1021/acs.langmuir.4c00498 (PMC11191699; doi:10.1021/acs.langmuir.4c00498)
Supplement: Supplementary file 1 — la4c00498_si_001.pdf [file la4c00498_si_001.pdf]

Impact of cations and framework on trapdoor behaviour: a study of dynamic and in-situ gas analysis

Dankun Yang[a], Huan V. Doan[a,b], Una O'Hara[c], Daniel Reed[d], Julian Hungerford[e], Jean-Charles Eloi[f], Natalie E. Pridmore[f], Paul F. Henry[g,h], Sebastien Rochat[f i] Mi Tian[j], Valeska P. Ting[a,b]*

a. Department of Mechanical Engineering, University of Bristol, Bristol BS8 1TR, UK
b. Research School of Chemistry, Australian National University, Canberra 2601, Australia
c. Department of Chemistry, University of Birmingham, Birmingham B15 2TT, UK
d. School of Metallurgy & Materials, University of Birmingham, Birmingham, B15 2TT, UK
e. Micromeritics Instrument Corp. Norcross GA, 30093, US
f. School of Chemistry, University of Bristol, Bristol BS8 1TS, UK
g. ISIS Pulsed Neutron & Muon Source, Rutherford Appleton Laboratory, Harwell Campus, Didcot, OX11 0QX, UK
h. Department of Chemistry - Ångström Laboratory, Lägerhyddsvägen 1, Box 538, SE-751 21 Uppsala, Sweden
i. School of Engineering Mathematics and Technology, University of Bristol, Bristol BS8 1TS, UK
j. Department of Engineering, University of Exeter, Exeter EX4 4QF, UK.

* v.ting@bristol.ac.uk, valeska.ting@anu.edu.au

1. Synthesis of CHAs and MERs

The raw material used for the synthesis of chabazite and merlinoite was zeolite Y (334413-100G, Aldrich) with a composition of (0.17 Na_2O: Al_2O_3: $8SiO_2$:500 H_2O).

For chabazite synthesis: Around 5g of zeolite Y **was dehydrated with an open surface** in a tube furnace with an N_2 flow at 450 °C for 6 hours, using a temperature ramp-up rate of 5 °C min^{-1}. The dehydrated zeolite (~4 g) was then mixed with 1 M KOH in a 1:9 solid-to-liquid ratio, by stirring. This mixture was then heated in a binder oven at 95 °C for 96 h. After that, the product was washed with deionised water using a centrifuge until the pH reached 7. The washed sample was dried at room temperature in air to obtain the parent chabazite (KNaCHA), which was confirmed by PXRD.

For merlinoite synthesis: Around 5g of zeolite Y **was wrapped in aluminium foil** and dehydrated in a tube furnace with an N_2 flow at 450 ^oC for 6 hours, using a temperature ramp-up rate of 5 ^oC min^{-1}. The dehydrated zeolite (~4.2 g) was then mixed with 1 M KOH in a 1:9 solid-to-liquid ratio, by stirring. The mixture was then heated in an oven at 95 ^oC for 96 h. After that, the product was washed with deionised water using a centrifuge till the pH reached 7. The washed sample was dried at room temperature in air to obtain the parent merlinoite (KMER), as confirmed by PXRD.

2. Additional results and information

2.1. SEM images

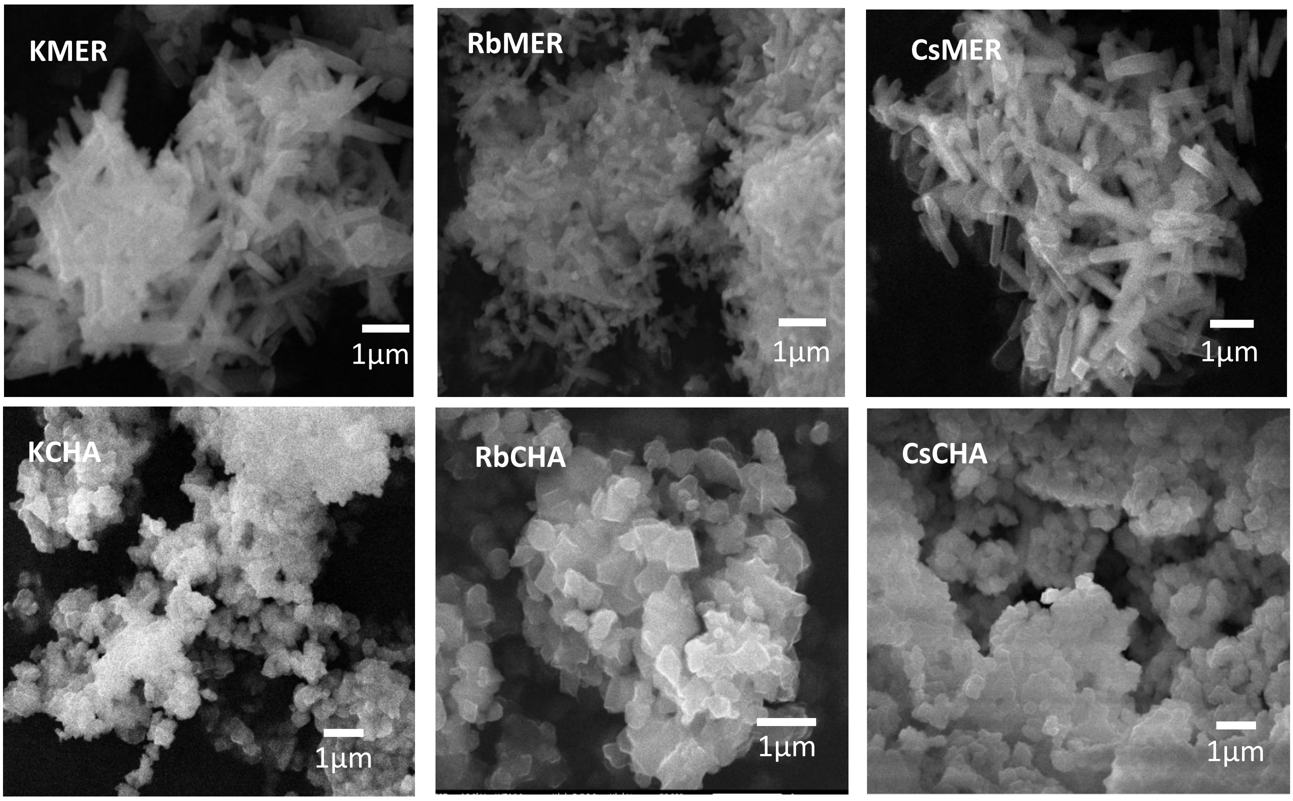

Figure S1: SEM images of all synthesised samples

2.2. Elemental concentration

Table S1: Elemental concentrations of all candidates and starting material from ICP-OES

Sample	Na ppm	Rb ppm	K ppm	Cs ppm	Al ppm	Si ppm
**KCHA**			2.2		2.2	2.8
**RbCHA**		4.8			1.9	2.3
**CsCHA**				**	2.1	2.6
**KMER**			2.4		2.3	2.9
**RbMER**		5.6			2.2	2.6
**CsMER**				**	1.9	2.6
**Y-zeolite**	0.3				1.8	2.9

ICP-OES was carried out on the synthesised zeolites (Table S1) to confirm the elemental concentration, but were hampered by limitations on the emission energy detected from the Group I elements. The ICP-OES response generated by Group I elements, especially Cs^+, is fairly weak and close to the wavelength of Ar, which leads to misleading results. Apart from that, the ICP-OES was not able to give the concentration for elements like oxygen, which has a high presence in zeolites. Moreover, for zeolites with a Si/Al ratio above 2, dissolving the samples can be an issue, due to the limited solubility of zeolites. As suggested by Hartman *et al.* [1], zeolites with a Si/Al ratio above 2, particularly the silicon component, may not completely dissolve in HCl, thus explaining the lower Si concentration observed compared to EDX results in all tested materials. Additionally, the wavelength emitted by Cs^+ closely resembles that emitted by Ar[2], resulting in inaccurate detection of Cs^+ concentration in all samples. However, elemental results for other elements are consistent with concentrations obtained from EDX analyses, and clear enrichment of the cations in the ion-exchanged synthesised frameworks can be seen when comparing the starting materials, as suggested by the EDX.

For example,: $\frac{Rb}{Al}\, in\, At\,\% = \frac{Rb\,(ppm)/85}{Al(ppm)/27}$ = 0.80 from ICP-OES in RbCHA and 0.81 in RbMER

In EDX $\frac{Rb}{Al}\, in\, At\,\% = \frac{Si/Al}{Si/Rb}$ =0.78 in RbCHA and 0.83 in RbMER

Also, for materials like zeolites that are not surface-enriched, the EDX results calculated from the average of the entire surface area can be representative and close to the results from ICP-OES (Nanoscale, 2022,14, 5915-5928; Environ Chem Lett 4, 235–238 (2006); Jordan Journal of Chemistry Vol. 7 No.4, 2012, pp. 383-391).

2.3. Pore size distribution from N_2 sorption of merlinoites and chabazites

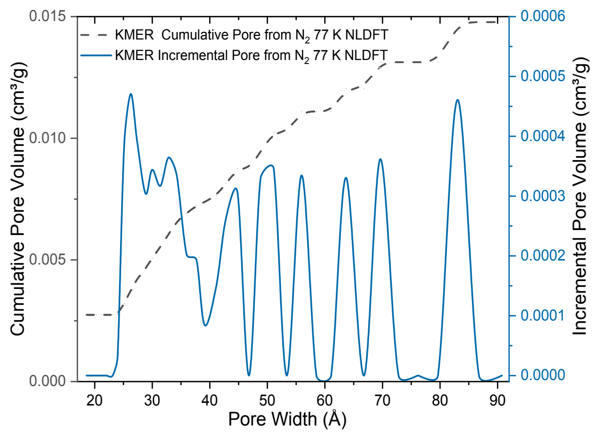

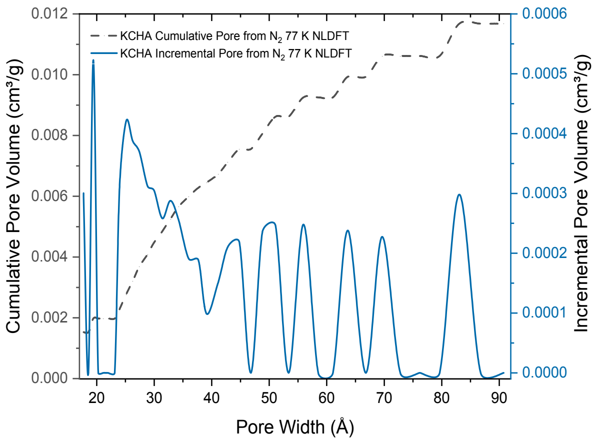

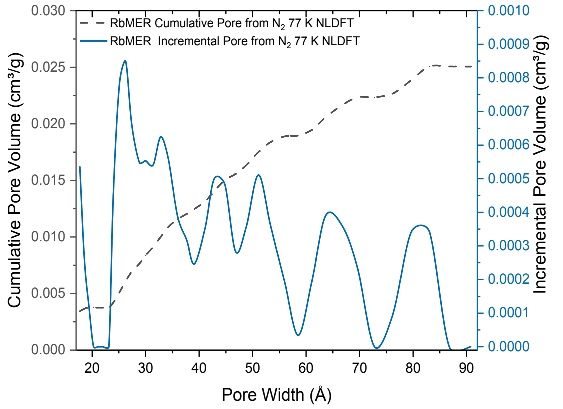

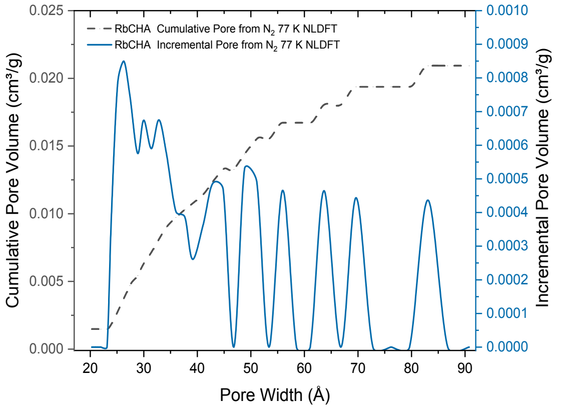

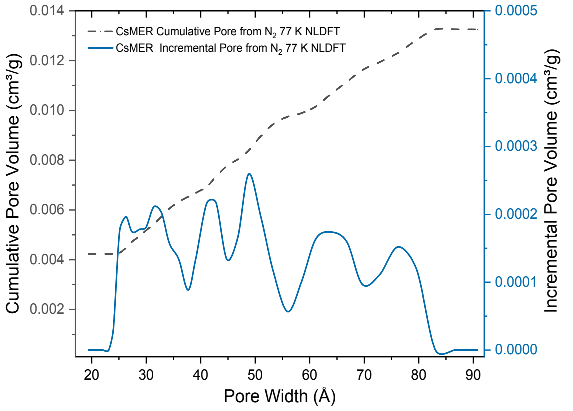

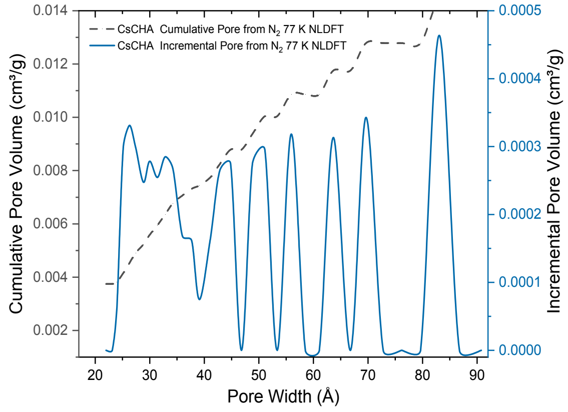

Figure S2: Pore size distribution of samples measured by N_2 sorption at 77K using NLDFT.

2.4. PXRD data and unit cell information

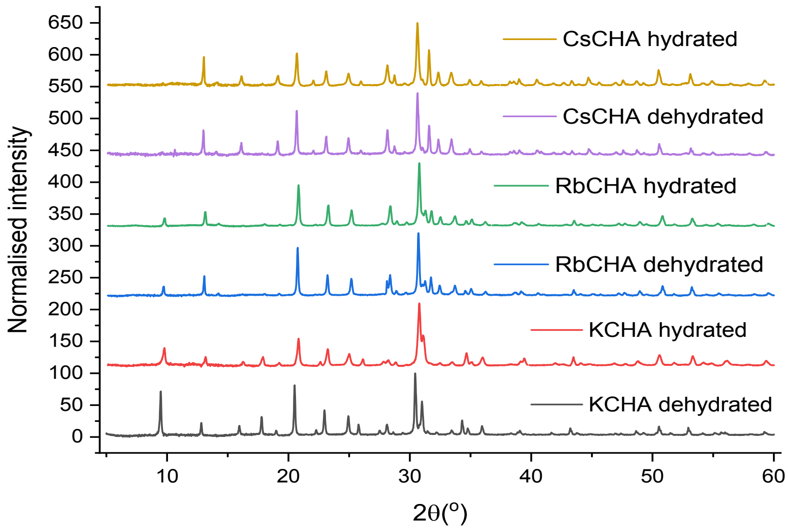

Figure S3: PXRD of samples before and after degassing under an Ar environment at room temperature.

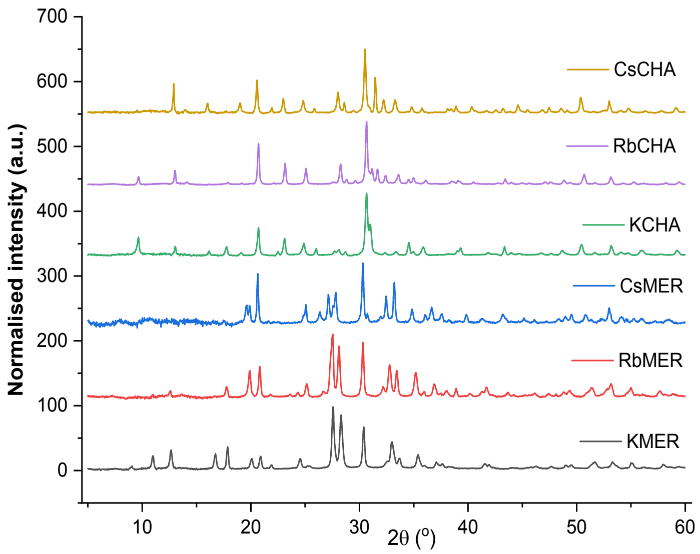

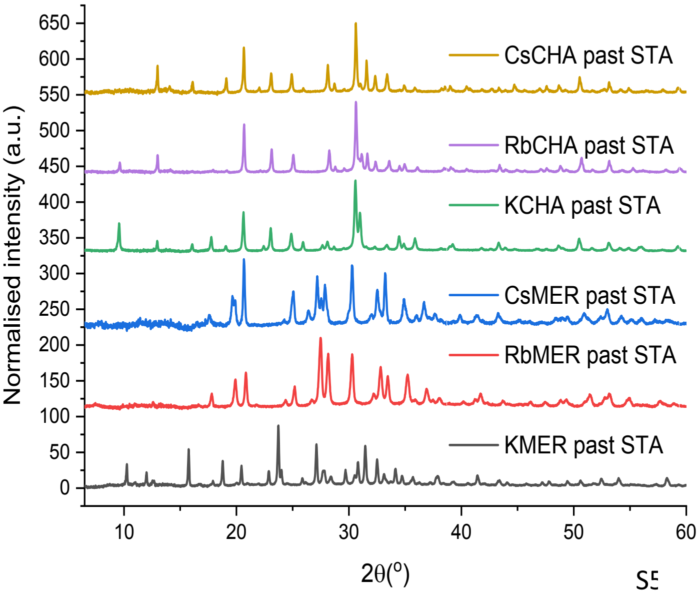

Figure S4: PXRD of samples before (left) and after STA (right) measurement in the atmosphere at room temperature.

2.5. Breakthrough raw data

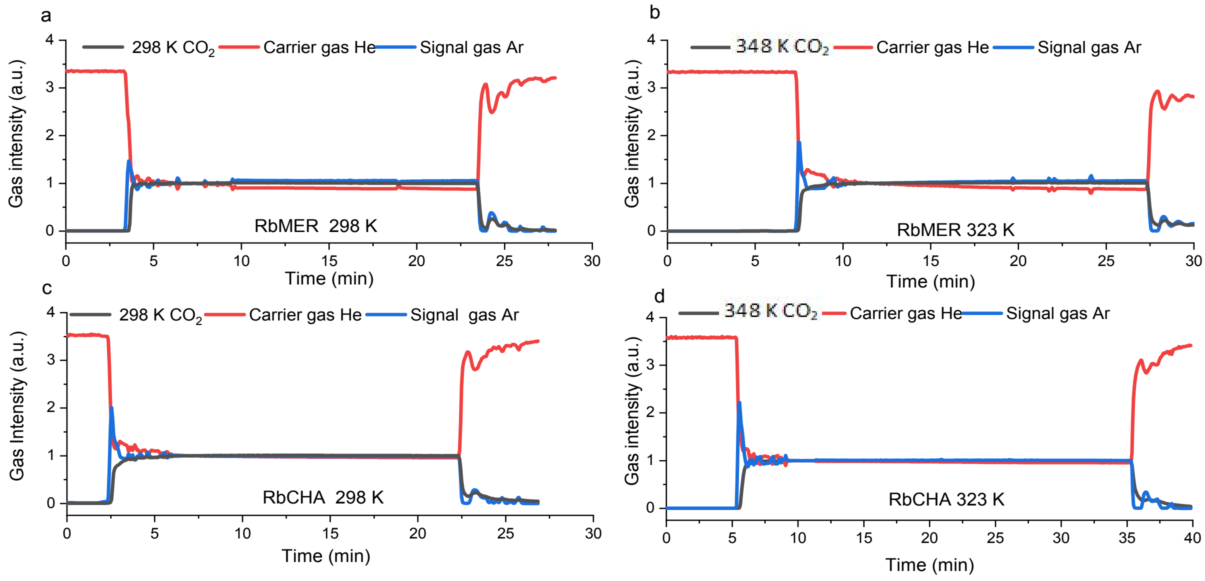

Figure S5: Breakthrough results at 2 bar for CO_2 with He as carrier gas (red) and Ar as a carrier gas (blue) a) RbMER at 298 K (black); b) RbMER at 348 K (black); c) RbCHA at 298 K (black); d) RbCHA at 348 K (black)

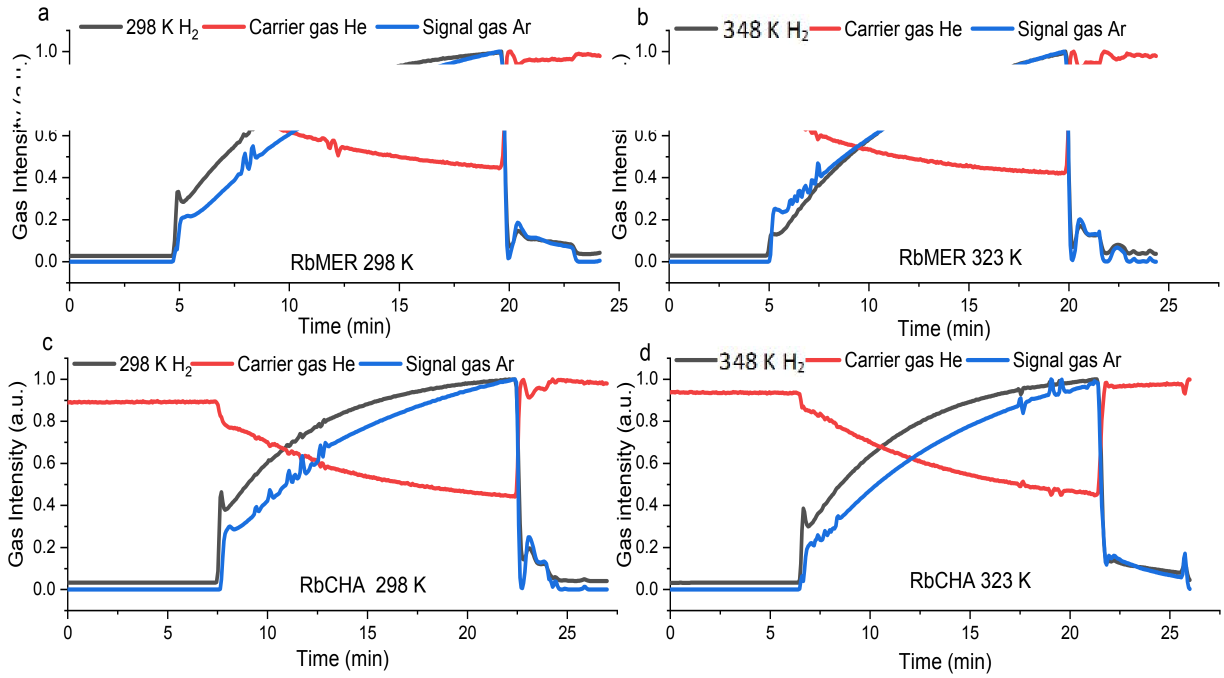

Figure S6: Breakthrough results at 2 bar for H_2 with He as carrier gas (red) and Ar as a carrier gas (blue) a) RbMER at 298 K (black); b) RbMER at 348 K (black); c) RbCHA at 298 K (black); d) RbCHA at 348 K (black).

2.6. In-situ PXRD results

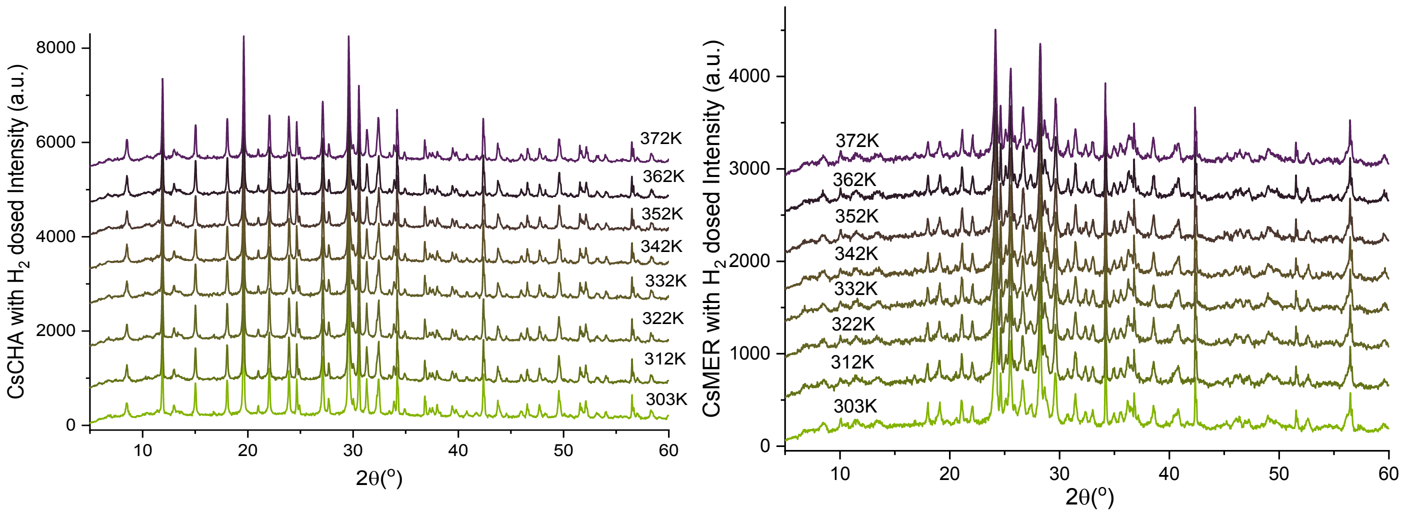

Figure S7: In-situ PXRD for candidates from room temperature up to 372 K under 1 bar H_2 flow.

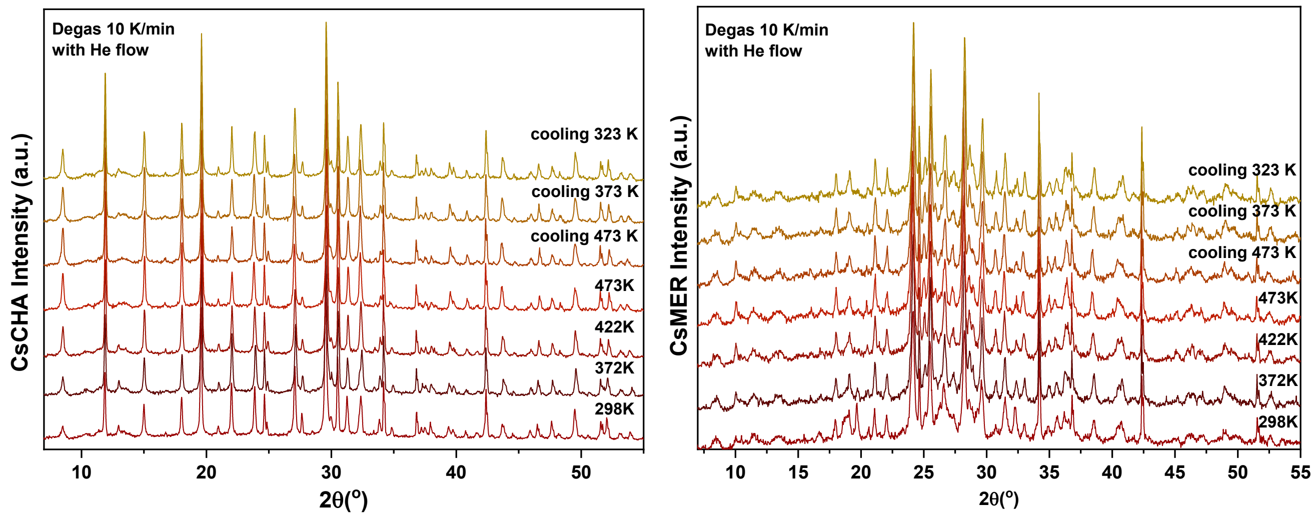

Figure S8: In-situ PXRD for candidates from room temperature up to 473 K with He flow.

2.7 Radiation stability of zeolites

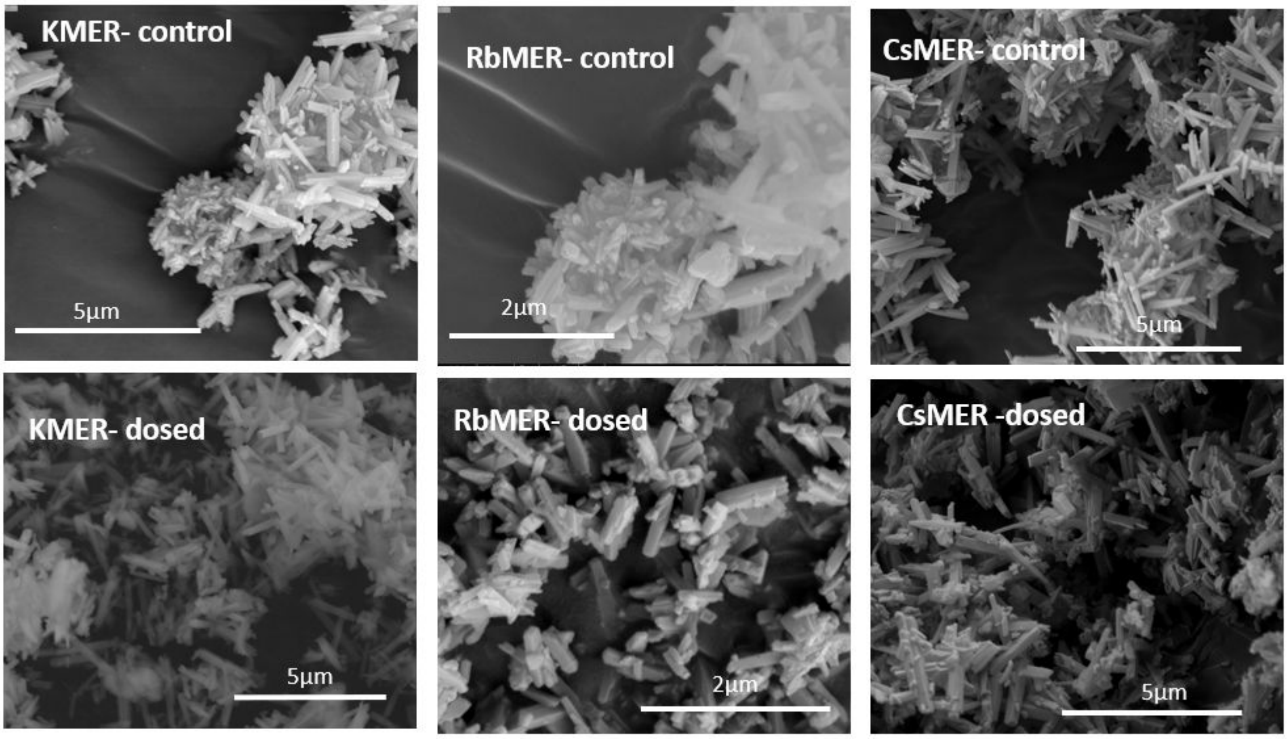

Figure S9: SEM images of MER-type zeolites exposed to Cs-137 vs control MER-type zeolites.

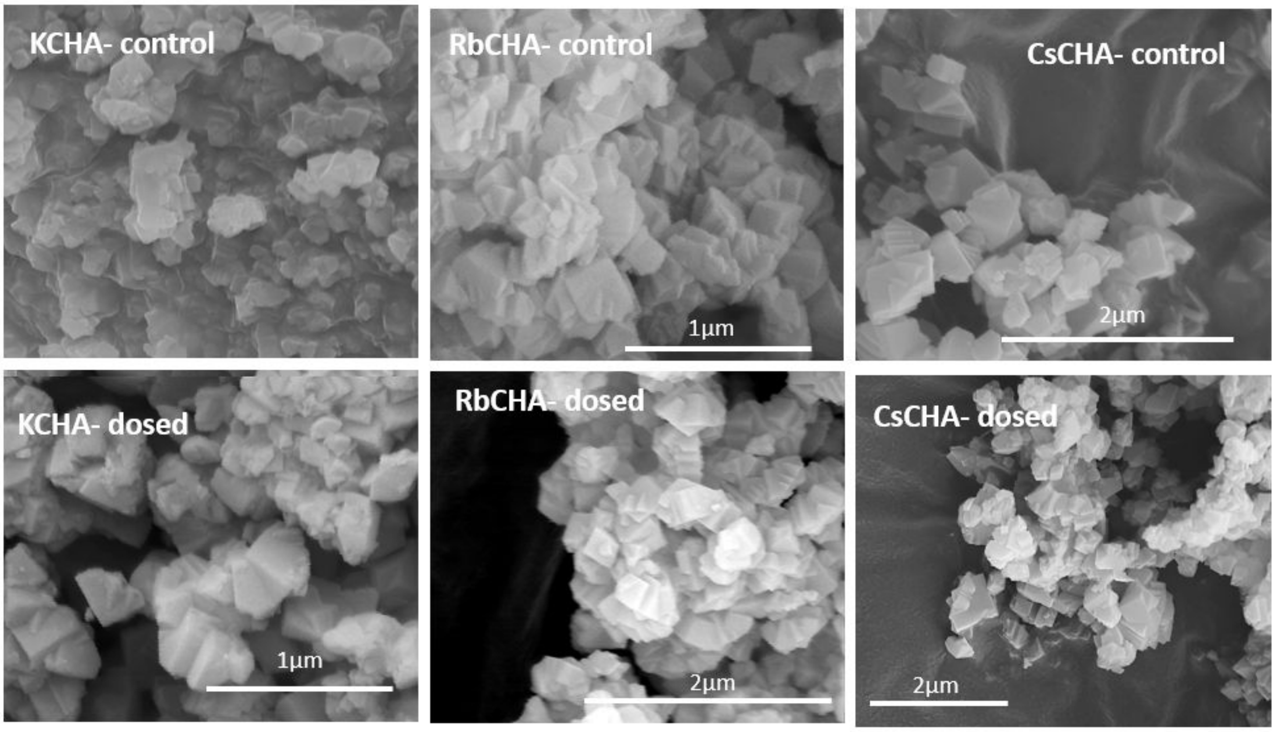

Figure S10: SEM images of CHA-type zeolites exposed to Cs-137 vs control CHA-type zeolites.

References

(1) Hartman, R. L.; Fogler, H. S. Understanding the Dissolution of Zeolites. *Langmuir* **2007**, *23* (10), 5477–5484. https://doi.org/10.1021/la063699g.

(2) Olesik, J. W. Elemental Analysis Using ICP-OES and ICP/MS. *Anal. Chem.* **1991**, *63* (1), 12A-21A. https://doi.org/10.1021/ac00001a001.
